# One‐ and Two‐Electron Transfer Oxidation of 1,4‐Disilabenzene with Formation of Stable Radical Cations and Dications

**DOI:** 10.1002/chem.202103715

**Published:** 2021-12-16

**Authors:** Yilin Chen, Zhikang Chen, Liuyin Jiang, Jiancheng Li, Yiling Zhao, Hongping Zhu, Herbert W. Roesky

**Affiliations:** ^1^ State Key Laboratory of Physical Chemistry of Solid Surfaces National Engineering Laboratory for Green Chemical Productions of Alcohols-Ethers-Esters College of Chemistry and Chemical Engineering Xiamen University Xiamen 361005 China; ^2^ Institut für Anorganische Chemie Georg-August-Universität 37077 Göttingen Germany

**Keywords:** 1,4-disilabenzene, dication, electron transfer, organometallic oxidant, radical cation

## Abstract

Electron‐transferable oxidants such as B(C_6_F_5_)_3_/*n*BuLi, B(C_6_F_5_)_3_/LiB(C_6_F_5_)_4_, B(C_6_F_5_)_3_/LiHBEt_3_, Al(C_6_F_5_)_3_/(*o*‐RC_6_H_4_)AlH_2_ (R=N(CMe_2_CH_2_)_2_CH_2_), B(C_6_F_5_)_3_/AlEt_3_, Al(C_6_F_5_)_3_, Al(C_6_F_5_)_3_/*n*BuLi, Al(C_6_F_5_)_3_/AlMe_3_, (CuC_6_F_5_)_4_, and Ag_2_SO_4_, respectively were employed for reactions with (L)_2_Si_2_C_4_(SiMe_3_)_2_(C_2_SiMe_3_)_2_ (L=PhC(N*t*Bu)_2_, **1**). The stable radical cation [**1**]^+.^ was formed and paired with the anions [*n*BuB(C_6_F_5_)_3_]^−^ (in **2**), [B(C_6_F_5_)_4_]^−^ (in **3**), [HB(C_6_F_5_)_3_]^−^ (in **4**), [EtB(C_6_F_5_)_3_]^−^ (in **5**), {[(C_6_F_5_)_3_Al]_2_(*μ*‐F)]^−^ (in **6**), [*n*BuAl(C_6_F_5_)_3_]^−^ (in **7**), and [Cu(C_6_F_5_)_2_]^−^ (in **8**), respectively. The stable dication [**1**]^2+^ was also generated with the anions [EtB(C_6_F_5_)_3_]^−^ (**9**) and [MeAl(C_6_F_5_)_3_]^−^ (**10**), respectively. In addition, the neutral compound [(L)_2_Si_2_C_4_(SiMe_3_)_2_(C_2_SiMe_3_)_2_][*μ*‐O_2_S(O)_2_] (**11**) was obtained. Compounds **2**–**11** are characterized by UV‐vis absorption spectroscopy, X‐ray crystallography, and elemental analysis. Compounds **2**–**8** are analyzed by EPR spectroscopy and compounds **9**–**11** by NMR spectroscopy. The structure features are discussed on the central Si_2_C_4_‐rings of **1**, [**1**]^+.^, [**1**]^2+^, and **11**, respectively.

## Graphical abstract

**Compounds [1]^+.^[R]^−^ and** [**1**]^2+^ ⋅ 2[R]^**−**^ have been prepared from the respective one‐ and two‐electron transfer reactions of 1,4‐disilabenzene (L)_2_Si_2_C_4_(SiMe_3_)_2_(C_2_SiMe_3_)_2_ (**1**) by using the transferable organometallic oxidants and characterization by X‐ray crystallography.

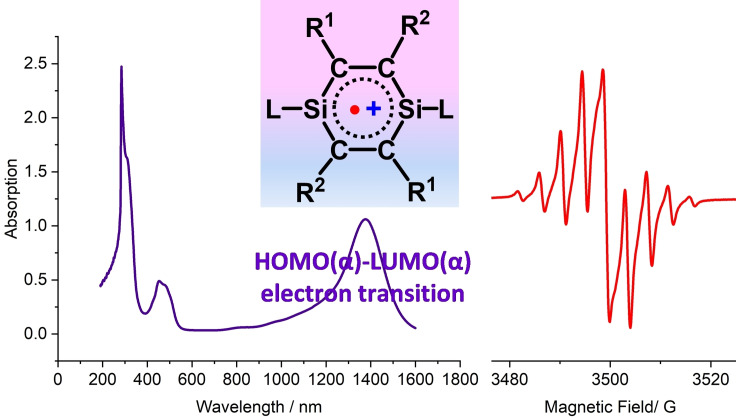

## Introduction

Most organic radicals are extremely reactive species and their synthesis and reactions have attracted great interest to chemists for over 120 years.^[1]^ In comparison to the carbon atom‐based radicals, the radicals of silicon congeners are in limited numbers available. The organosilicon‐based radicals are acting as reactive intermediates in numerous organic and organometallic transformations.^[2]^ Most of them have been detected using spectroscopic investigations.[^3^, ^4^] Only recently, a number of organosilicon radicals have been synthesized and characterized by X‐ray single‐crystal structural analysis.[^5^, ^6^, ^7^, ^8^, ^9^, ^10^, ^11^, ^12^, ^13^]

**Scheme 1:**
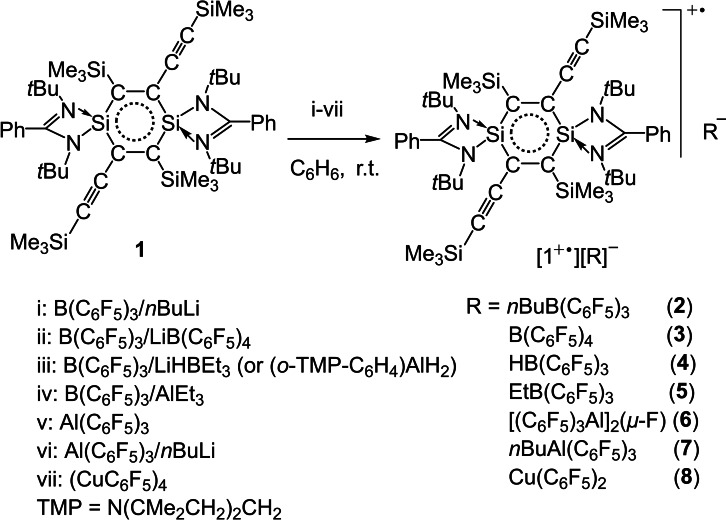
One‐electron transfer oxidation reactions of **1** to form compounds **2**–**8**.

**Scheme 2:**
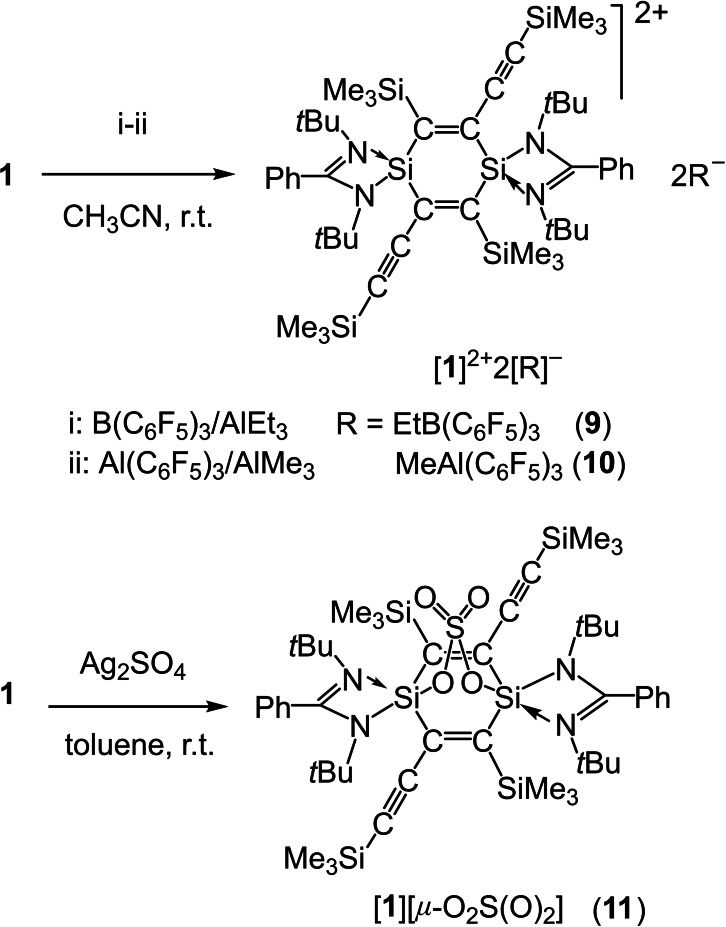
Two‐electron transfer oxidation reactions of **1** to form compounds **9**–**11**.

Benzene and its derivatives are a pivotally important class of basic organic molecules, which feature a 6π‐electron delocalization over the C_6_ ring and exhibit considerable stability due to the Hückel aromatic nature.^[14]^ Radicals, cations, anions, and radical cations and anions of benzene and the derivatives have been studied and applied widespread.^[15]^ The silicon‐substituted benzene analogs have been predicted in theory,^[16]^ and later detected spectroscopically in the gas phase and/or in low‐temperature matrices.^[17]^ In the year 2000 the first stable organo‐silicon compounds of this type have been isolated. So far the following species have been characterized and structurally authenticated: (H)_5_C_5_Si(tbt) (tbt=2,4,6‐[CH(SiMe_3_)_2_]_3_C_6_H_2_) (2000),^[18]^ 1,2‐disilabenzene (Ph)_2_(H)_2_C_4_Si_2_(R)_2_ (R=Si*i*Pr[CH(SiMe_3_)_2_]_2_ (2007);^[19]^ (R′)_2_(H)_2_C_4_Si_2_(Bbt)_2_ (R′=H, SiMe_3_, Ph; Bbt=2,6‐[CH(SiMe_3_)_2_]_2_‐4‐C(SiMe_3_)_3_C_6_H_2_) (2010),^[20]^ hexasilabenzene (tip)_2_Si_2_Si_2_Si_2_(tip)_4_ (tip=2,4,6‐*i*Pr_3_C_6_H_2_) (2010),^[21]^ and 1,4‐disilabenzene (Ph)_4_C_4_Si_2_(L)_2_ (L=PhC(N*t*Bu)_2_) (2010).^[22]^ However, their radicals and ions were yet not known. More recently we prepared a 1,4‐disilabenzene (L)_2_Si_2_C_4_(SiMe_3_)_2_(C_2_SiMe_3_)_2_ (**1**), and further obtained [**1**]^+.^[B(C_6_F_5_)_4_]^−^ and [**1**]^2+^(OSO_2_CF_3_)^2−^ using oxidation reactions.^[23]^ [**1**]^+^ represents the first example of the radical cation while [**1**]^2+^ exhibits a dication among the organosilicon benzene analogs.[^18^, ^19^, ^20^, ^21^, ^22^] Compound **1** is able to proceed by electron transfer basically contributing to stabilization of the organic auxiliary at the two Si atoms of the central C_4_Si_2_ ring. The selection of the electron transferable oxidant appears pivotal in reaction to form the anion(s) in pairing with either [**1**]^+.^ or [**1**]^2+^. Nevertheless, study on this chemistry is rare. The suitable oxidant is disclosed in a limited number. Herein, we discovered B(C_6_F_5_)_3_, Al(C_6_F_5_)_3_, and (CuC_6_F_5_)_4_, respectively as useful organometallic oxidants in reaction with **1** and [**1**]^+.^ was produced through a single electron transfer and [**1**]^2+^ by two‐electron oxidation (Schemes 1 and 2). Then an investigation allows us to discuss the structural features of the central Si_2_C_4_‐rings of **1**, [**1**]^+.^, and [**1**]^2+^, respectively in detail. Herein, we present a study on the reactions, where the products are characterized by X‐ray crystallography, UV‐vis absorption spectroscopy, electron paramagnetic resonance (EPR) spectroscopy, elemental analysis, and/or NMR spectroscopy.

## Results and Discussion

### One‐electron transfer oxidation of 1

We screened to use B(C_6_F_5_)_3_ for the reaction with compound **1**. At room temperature, the addition of C_6_H_6_ into a solid mixture of **1** (dark purple) and equivalent B(C_6_F_5_)_3_ (off‐white) led to a quick color change into a deep brown. The EPR measurement showed the resonances implying the formation of the radical species.^[23]^ However, this reaction mixture produced an oily mass after ca. 10 min, from which isolation of the pure product was not successful. By a number of try‐outs, the addition of *n*BuLi resulted in a pure product. Thus, mixing of B(C_6_F_5_)_3_ and **1** in a 1 : 1 molar ratio in C_6_H_6_ at room temperature followed by the addition of *n*BuLi (*n*‐hexane solution) smoothly resulted in compound [**1**]^+.^[*n*BuB(C_6_F_5_)_3_]^−^ (**2**). Compound **2** was isolated as yellow crystals in 50 % yield after the reaction solution was stored for 12 h without any disturbance. Wildgoose and coworkers reported the reaction of B(C_6_F_5_)_3_ and Cp*_2_Co in CH_2_Cl_2_ (or 1,2‐F_2_C_6_H_4_) to produce a mixture of anions [HClB(C_6_F_5_)_2_]^−^, [Cl_2_B(C_6_F_5_)_2_]^−^, [ClB(C_6_F_5_)_3_]^−^, [HB(C_6_F_5_)_3_]^−^, and [B(C_6_F_5_)_4_]^−^, each in pairing with (Cp*_2_Co)^+^ on the basis of NMR and MS characterization.^[24]^ The B(C_6_F_5_)_3_ was able to abstract one electron^[25]^ from Cp*_2_Co to generate [B(C_6_F_5_)_3_]^−.^ and (Cp*_2_Co)^+^. Additional products resulted from solvent by H or Cl atom abstraction to give [HB(C_6_F_5_)_3_]^−^ and [ClB(C_6_F_5_)_3_]^−^ followed with the group exchange reactions. Accordingly, we suggest that in the formation of **2** B(C_6_F_5_)_3_ gained an electron from **1** to yield [B(C_6_F_5_)_3_]^−.^ and [**1**]^+.^. The [B(C_6_F_5_)_3_]^−.^ could snatch one H atom from the solvent media (C_6_H_6_ or *n*‐hexane) to generate [HB(C_6_F_5_)_3_]^−^. The latter reacted with *n*BuLi to produce [*n*BuB(C_6_F_5_)_3_]^−^, an anion in pairing with [**1**]^+.^, and HLi. We attempted to identify the possible species formed from the H atom‐abstracted solvent molecule but were not successful.^[26]^ However, we were able to detect the HLi‐like species through IR spectral measurement.^[27]^ It is worth mentioning that no reaction happened when using *n*BuLi alone with **1**. The B(C_6_F_5_)_3_ is indeed the electron transfer oxidant rather than the *n*BuLi. The *n*BuLi matches well to form the stable isolated product through exchange reaction. Furthermore, a premixing of B(C_6_F_5_)_3_ and *n*BuLi was not working for oxidation of **1**. Mostly, Li[*n*BuB(C_6_F_5_)_3_] was quickly produced that shuts down any electron transfer.

Furthermore, we used B(C_6_F_5_)_3_/LiB(C_6_F_5_)_4_ instead and prepared compound [**1**]^+.^[B(C_6_F_5_)_4_]^−^ (**3**) in C_6_H_6_. Compound **3** was separated as yellow crystals with a yield of 42 % and it is the same product as that obtained from the reaction of **1** with [Ph_3_C]^+^[B(C_6_F_5_)_4_]^−^.^[23]^ The [Ph_3_C]^+^ has been known as an electron transfer oxidant,^[25a]^ which gained an electron from **1** to produce [Ph_3_C ⋅ ] as the side product and then the [B(C_6_F_5_)_4_]^−^ turned to be the non‐coordinating anion. Herein B(C_6_F_5_)_3_ reacted by one‐electron transfer followed with the H atom‐abstraction from C_6_H_6_ to give [**1**]^+.^ and [HB(C_6_F_5_)_3_]^−^. [HB(C_6_F_5_)_3_]^−^ reacted further with Li[B(C_6_F_5_)_4_] to form [B(C_6_F_5_)_4_]^−^, as the counterpart of [**1**]^+.^, and Li[HB(C_6_F_5_)_3_]. The LiB(C_6_F_5_)_4_ alone was tested and shows no reaction with **1**.

Moreover, we employed B(C_6_F_5_)_3_/LiBHEt_3_ for the reaction and finally obtained compound [**1**]^+.^[HB(C_6_F_5_)_3_]^−^ (**4**) from a mixture of C_6_H_6_ and THF. Compound **4** was isolated as block‐like crystals with green color and a yield of 47 %. [HB(C_6_F_5_)_3_]^−^ is herein the target anion, and this may prove its existence as an intermediate in the production of either **2** or **3**. Alternatively to B(C_6_F_5_)_3_/(*o*‐RC_6_H_4_)AlH_2_ ^[28]^ (R=N(CMe_2_CH_2_)_2_CH_2_, 60 % yield) **4** too is formed in C_6_H_6_.

In addition, reaction of **1** with B(C_6_F_5_)_3_/AlEt_3_ resulted in [**1**]^+.^[EtB(C_6_F_5_)_3_]^−^ (**5**). Compound **5** was isolated as yellow crystals (41 %). It was interesting to see that AlEt_3_ alone was capable of oxidizing **1** as well as indicated by a color change into typically brown, although isolation of a pure product failed. Similar property has been reported by Stephan et al. where Al(C_6_F_5_)_3_ was used for reactions.[^25c^, ^29^] Nonetheless, on the basis of formation of the [EtB(C_6_F_5_)_3_]^−^ anion, B(C_6_F_5_)_3_ might dominantly behave as the one‐electron oxidant, while AlEt_3_ reacted with an H−Et^−^ exchange of [HB(C_6_F_5_)_3_]^−^. Compound (*o*‐RC_6_H_4_)AlH_2_ showed no reactivity. The oxidation activity might be inhibited due to an intramolecular regio‐positioned N→Al coordination.^[28]^

We then turned to Al(C_6_F_5_)_3_/*n*BuLi and obtained compound [**1**]^+.^[*n*BuAl(C_6_F_5_)_3_]^−^ (**7**, 67 %, yellow crystals). It was surprising to find that Al(C_6_F_5_)_3_ alone yielded compound [**1**]^+.^{[(C_6_F_5_)_3_Al]_2_(*μ*‐F)}^−^ (**6**) as yellow crystals in 15 % yield. Obviously, Al(C_6_F_5_)_3_ abstracted one electron from **1** to form [Al(C_6_F_5_)_3_]^−.^ and [**1**]^+.^. In the absence of *n*BuLi, the [Al(C_6_F_5_)_3_]^−.^ snatched an F atom from the C_6_F_5_ group^[30]^ to give [FAl(C_6_F_5_)_3_]^−^ that ligated another molecule of Al(C_6_F_5_)_3_ to form a non‐coordinated anion {[(C_6_F_5_)_3_Al]_2_(*μ*‐F)}^−^. With the presence of *n*BuLi, the [Al(C_6_F_5_)_3_]^−.^ was prone to abstract one H atom from the solvent to generate [HAl(C_6_F_5_)_3_]^−^.^[31]^ In addition, during the reaction an H^−^‐Bu^−^ exchange with *n*BuLi occurred.

Finally, we tried (CuC_6_F_5_)_4_ as the oxidant considering close oxidation ability between Cu^+^ and Ag^+^.^[32]^ The reaction was accomplished using **1** with (CuC_6_F_5_)_4_ in a molar ratio of 2 : 1 in C_6_H_6_ at room temperature. Compound [**1**]^+.^[(C_6_F_5_)_2_Cu]^−^ (**8**) was obtained as yellow‐green crystals in a yield of 54 %. The CuC_6_F_5_ received one electron from **1** to form Cu, C_6_F_5_^−^, and [**1**]^+.^, while the C_6_F_5_^−^ coordinated to anther molecule of CuC_6_F_5_ to give [(C_6_F_5_)_2_Cu]^−^ as the final counterion. A Cu mirror was observed after the reaction.

### Two‐electron transfer oxidation of 1

Previously, we prepared compound [**1**]^2**+**^ ⋅ 2[OSO_2_CF_3_]^−^ from **1** and AgOSO_2_CF_3_ in a 1 : 2 molar ratio in MeCN where two‐electron transfer oxidation was observed.^[23]^ The experiments by screening the number of solvents indicate that C_6_H_6_ is better for obtaining compounds **2**–**8**, while CH_3_CN is suitable for gaining [**1**]^2**+**^ ⋅ 2[OSO_2_CF_3_]^−^. Compound **5** was actually obtained by using C_6_H_6_, where a quick color change into a deep brown was observed after mixing B(C_6_F_5_)_3_/AlEt_3_ with **1** and **5** started to precipitate from the solution. However, this reaction changes, when conducted in CH_3_CN. Then, mixing of B(C_6_F_5_)_3_/AlEt_3_ with half equivalent of **1** in CH_3_CN exhibited the solution color change into a deep brown. However, instead of the precipitation of **5** further color change into light yellow and finally colorless occurred within 5 h, and compound [**1**]^2**+**^ ⋅ 2[EtB(C_6_F_5_)_3_]^−^ (**9**, colorless block crystals, 20 %) was obtained. This indicates that the B(C_6_F_5_)_3_/AlEt_3_ performs well with either one‐ or two‐electron transfer oxidation of **1**, and the reactions are depending on the solvent. Similarly, the reaction of Al(C_6_F_5_)_3_/AlMe_3_ (1 : 1 ratio) with half equivalent of **1** in CH_3_CN resulted in compound [**1**]^2+^ ⋅ 2 [MeAl(C_6_F_5_)_3_]^−^ (**10**, colorless crystals, 33 % yield, obtained by storing the reaction solution at −20 °C for 24 h). We also tried reactions in CH_3_CN in an attempt to obtain **2**–**4** and **6**–**8** but were not able to isolate the pure products. Obviously, the selection of the oxidants of B(C_6_F_5_)_3_/AlEt_3_ or of B(C_6_F_5_)_3_/AlMe_3_ is crucial. Finally, we carried out the reaction with one equivalent each of **1** and Ag_2_SO_4_ in toluene where we observed gradual dissolving and reacting of Ag_2_SO_4_. 2 h later, a silver mirror was formed. Picking up the colorless solution and storing it at −20 °C for 24 h resulted in [(L)_2_Si_2_C_4_(SiMe_3_)_2_(C_2_SiMe_3_)_2_][*μ*‐O_2_S(O)_2_] (**11**, colorless crystals, 80 % yield, Scheme 2).

### Spectroscopic characterization

Compounds **2**–**11** were isolated as crystals of X‐ray diffraction quality. They are sensitive to air and moisture. Compounds **2**–**8** are radicals and are studied by electron paramagnetic resonance (EPR). Compounds **9** and **10** are ionic in nature and compound **11** is neutral and they are performed by NMR analysis. Compounds **2**–**11** are investigated by UV‐vis spectroscopy.

**Figure 1:**
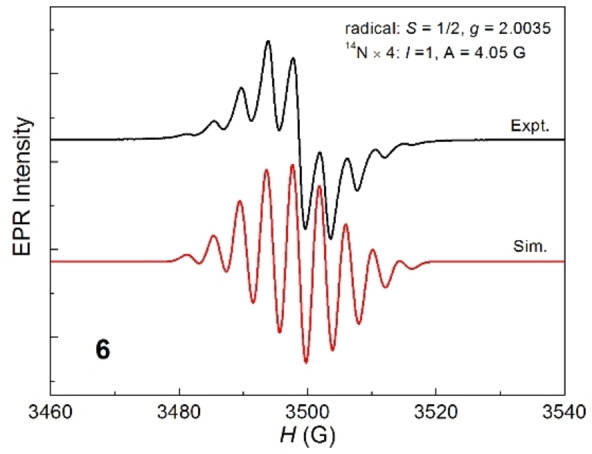
EPR spectra of compound **6** in toluene at 298 K with the simulated result.

**Figure 2:**
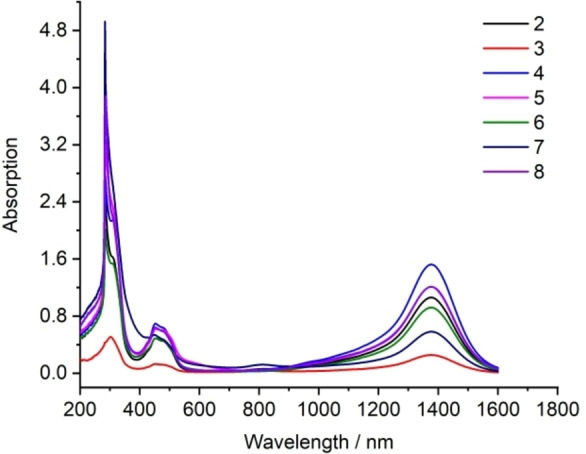
UV‐vis absorption spectra of compounds **2**–**8** measured in toluene at room temperature.

The EPR spectra of complexes **2**–**8** all exhibit well‐resolved signals consisting of nine resonances (Figures 1 and S15–S20). These signals are centered at a close *g* value of 2.0038 due to the same [**1**]^+.^ cation in each of **2**–**8**. These data suggest delocalization of the one‐electron over the central C_4_Si_2_ ring of [**1**]^+.^. The UV‐vis absorption spectra of **2**–**8** exhibit each the strong long‐wavelength absorption ranging within 1200–1600 nm (Figure 2), which is attributed to HOMO(α)‐LUMO(α) electron transition (*f*=0.16) for [**1**]^+.^ in each of **2**–**8** we discussed before.^[23]^ Compounds **9**–**11** were exposed to UV‐vis spectroscopy but show no absorption at the visible‐light region (Figure S21). This indicates no electron delocalization over the C_4_Si_2_‐ring in [**1**]^2+^ of **9** and **10** as well as in **11**, markedly unlike those with the two electrons in **1** and one electron in [**1**]^+.^ of **2**–**8**.

The ^1^H, ^13^C, and ^29^Si NMR data are recorded for compound **11**. However only ^1^H and ^19^F data were obtained for compounds **9** and **10** due to low solubility (Figures S22–S30). These data indicate the compositions and structures of **9**–**11** in line with those analysed by the X‐ray crystal structural analysis (Figures 4, 11, and S12–S14).

### X‐ray crystallographic characterization

**Figure 3:**
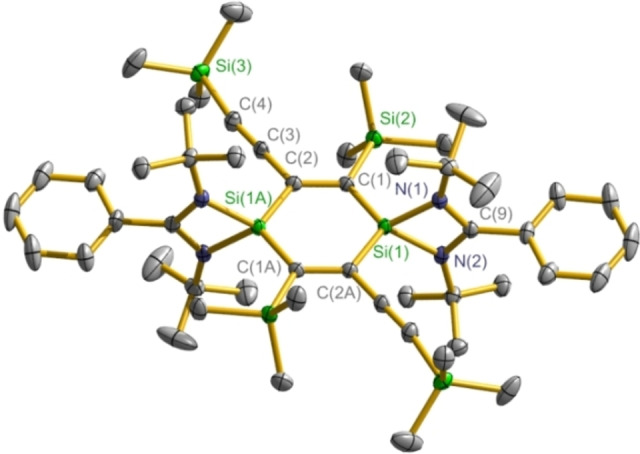
Crystal structure of [**1**]^+.^ in **6** with thermal ellipsoids at the 50 % probability level. Hydrogen atoms are omitted for clarity.

**Figure 4:**
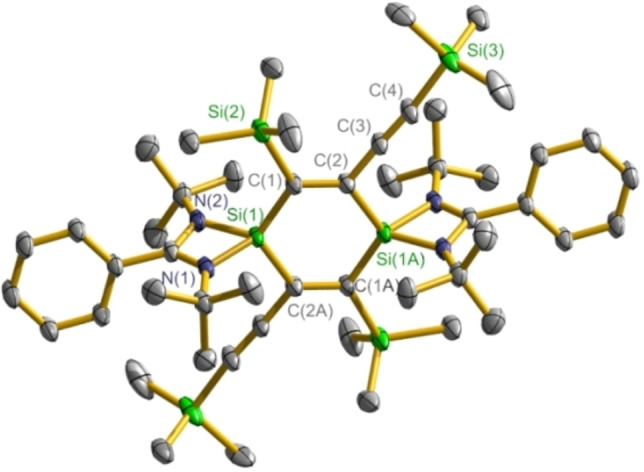
Crystal structure of [**1**]^2**+**^ in **10** with thermal ellipsoids at the 50 % probability level. Hydrogen atoms are omitted for clarity.

**Figure 5:**
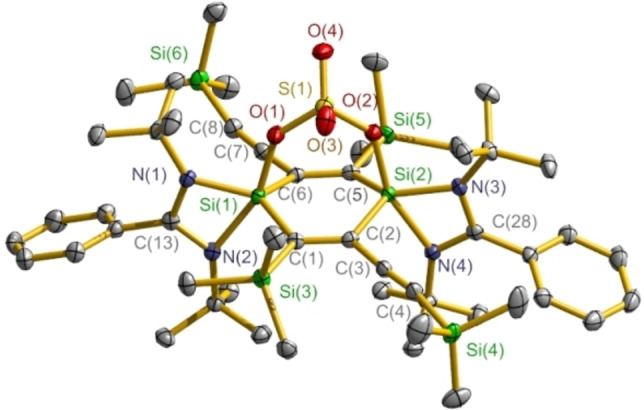
Crystal structure of **11** with thermal ellipsoids at the 50 % probability level. Hydrogen atoms are omitted for clarity.

The molecular structures of compounds **2** and **4**–**11** are disclosed by X‐ray single‐crystal diffraction study; the structure of compound **3** has been reported in our group.^[23]^ The structure determination reveals that compounds **2** and **4**–**8** all contain the radical cation [**1**]^+.^ (Figures 3, S1, S3, S5, S8, and S10). In pairing with [**1**]^+.^, the intermolecular separated anion is disclosed as [*n*BuB(C_6_F_5_)_3_]^−^ for **2** (Figure S2), [HB(C_6_F_5_)_3_]^−^ for **4** (Figure S4), [EtB(C_6_F_5_)_3_]^−^ for **5** (Figure S6), {[(C_6_F_5_)_3_Al]_2_(*μ*‐F)}^−^ for **6** (Figure S7), [*n*BuAl(C_6_F_5_)_3_]^−^ for **7** (Figure S9), and [Cu(C_6_F_5_)_2_]^−^ for **8** (Figure S11), respectively. Compounds **9** and **10** both consist of the dication [**1**]^2**+**^ (Figures 4 and S12), with the two standalone counterions of [EtB(C_6_F_5_)_3_]^−^ for **9** (Figure S13) and [MeAl(C_6_F_5_)_3_]^−^ for **10** (Figure S14). Compound **11** is a neutral species showing a *μ*‐O_2_S(O)_2_ bridge bond at the two Si atoms of the C_4_Si_2_ ring (Figure 5).

Table 1 summarizes the important bond parameters inside the C_4_Si_2_ and CN_2_Si rings of **1** and [**1**]^+.^ (at each of **2**–**8**) and Table 2 records those of [**1**]^2+^ at each of **9** and **10**. Also listed are the Wiberg bond indexes (WBIs) previously calculated for discussion.^[23]^ It is clearly seen that inside the C_4_Si_2_ rings, the C−C bond lengths are averaged into 1.446(2) for **1**, 1.401(5)–1.414(3) for [**1**]^+.^, and 1.368(3)–1.371(2) Å for [**1**]^2**+**^. The WBIs calculations indicate the related bond orders of 1.35, 1.53, and 1.73, respectively. To be correlated, the Si−C bond distances are 1.798(1) (for **1**), 1.817(2)–1.826(2) (for [**1**]^+.^), and 1.854(3)–1.861(2) Å (for [**1**]^2**+**^), with the WBIs of 1.23, 1.12, and 1.01. Meanwhile, in the CN_2_Si rings the Si−N bond lengths appear 1.845(1) for **1**, 1.807(3)–1.815(2) for [**1**]^+.^, and 1.778(2)–1.782(1) Å for [**1**]^2**+**^, whereas the C−N bond distances exhibit little changes (1.336(2) for **1**, 1.338(2)–1.342(3) for [**1**]^+.^, and 1.346(4)–1.349(2) Å for [**1**]^2**+**^).

**Table 1:** Data for key bond lengths (Å), least square planes (Δ, Å), and calculated Wiberg bond indexes (WBIs) of **1** together with those of [**1**]^+.^ in **2**–**8**.

Sample	Bond	1	[**1**]^+.^ in **2**	[**1**]^+.^ in **3**	[**1**]^+.^ in **4**	[**1**]^+.^ in **5**	[**1**]^+.^ in **6**	[**1**]^+.^ in **7**	[**1**]^+.^ in **8**
C4Si2 cycle^[a]^	C4Si2 cycle^[a]^	C−C WBI Si−C WBI Δ	1.446(2) 1.35 1.798(1) 1.23 0.0078	1.401(5) 1.823(4) 0.0154	1.408(3) 1.822(2) 0.0222	1.401(3) 1.826(2) 0.0360 0.0308	1.402(3) 1.53 1.820(2) 1.12 0.0017 0.0156	1.406(2) 1.826(2) 0.0192	1.414(3) 1.817(2) 0.0043 0.0097
CN2Si cycle^[a^	Si−N C−N Δ	1.845(1) 1.336(2) 0.0264	1.807(3) 1.340(5) 0.0300 0.0265	1.812(2) 1.342(3) 0.0277	1.812(2) 1.340(3) 0.0027 0.0019	1.807(2) 1.340(2) 0.0252 0.0316	1.815(2) 1.338(2) 0.0143	1.811(2) 1.341(3) 0.0280 0.0321	1.811(2) 1.341(3) 0.0255

[a] Average bond data.

**Table 2:** Data for key bond lengths (Å), least‐square planes (Δ, Å), and calculated Wiberg bond indexes (WBIs) of [**1**]^2+^ in **9** and **10** together with those of [**1**] in **11**.

Sample	Bond	[**1**]^2**+**^ in **9**	[**1**]^2**+**^ in **10**	[**1**] In **11**
C_4_Si_2_ cycle^[a]^	C−C WBI Si−C WBI Δ	1.371(2) 1.73 1.861(2) 1.01 0.0100	1.368(3) 1.854(3) 0.0078	1.367(2) 1.889(2) 0.1566
CN_2_Si cycle^[a]^	Si−N C−N Δ	1.782(1) 1.349(2) 0.0065	1.778(2) 1.346(4) 0.0266	1.905(2) 1.335(2) 0.0370 0.0205

[a] Average bond data.

**Scheme 3:**
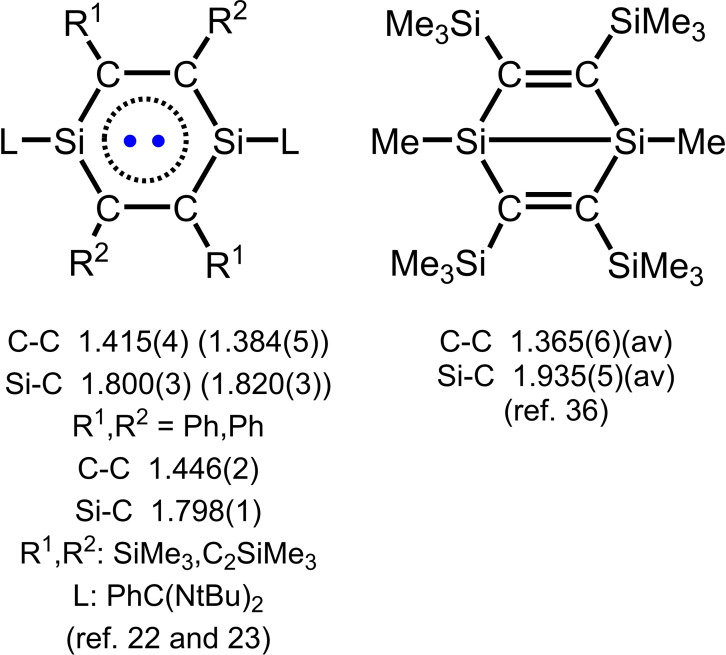
Schematic structures of 1,4‐disilabenzene and 1,4‐disila(Dewar‐benzene) with the related bond lengths over the central rings.

**Scheme 4:**
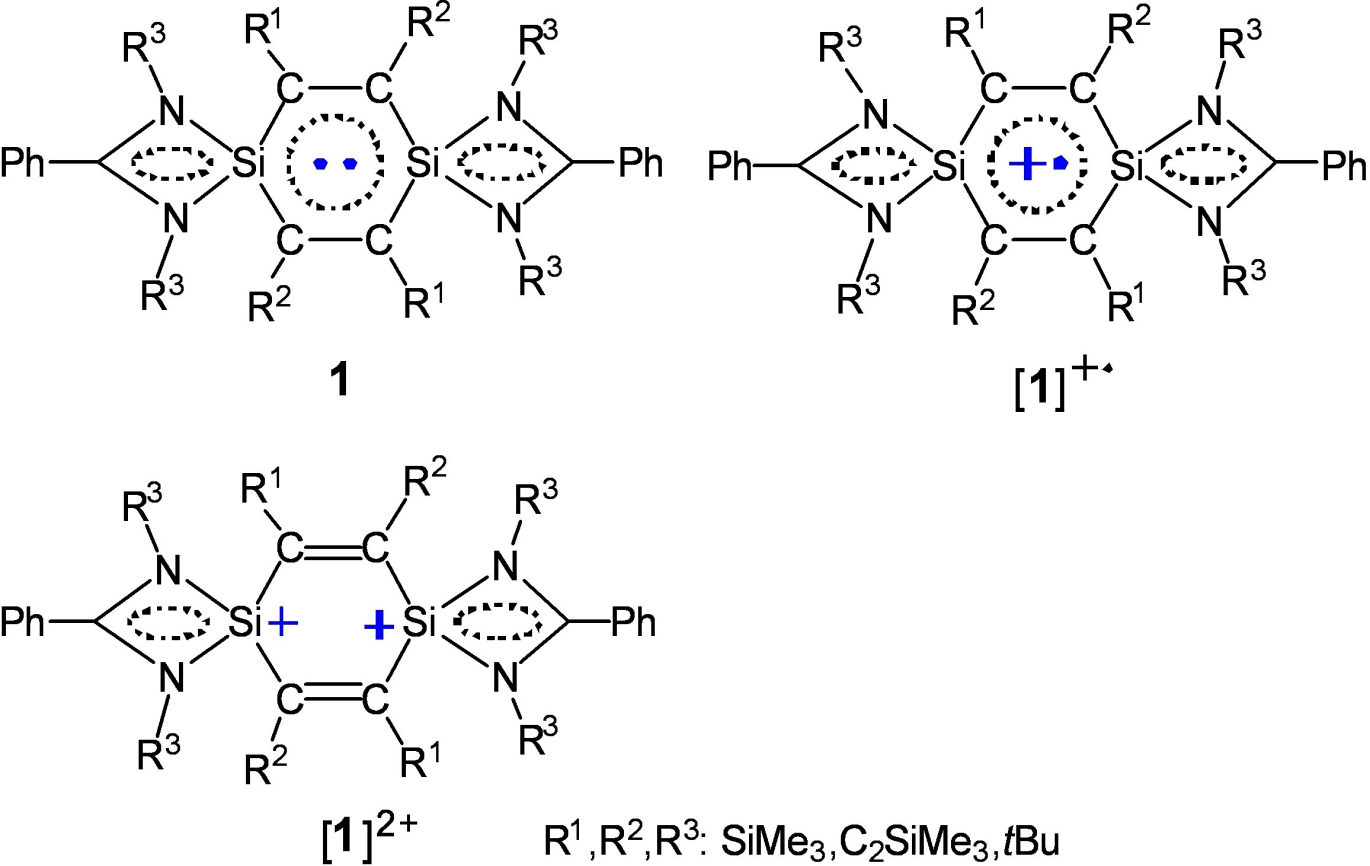
Schematic electronic structures of **1**, [**1**]^+.^ (in **2**–**8**), and [**1**]^2+^ (in **9** and **10**).

Compound **1** has been well proved to be the open‐shell singlet diradical‐like compound (L)_2_Si_2_C_4_(Ph)_4_.^[22]^ The electron delocalization occurs over the C_4_Si_2_ ring as well as the CN_2_Si cycle. Ando and coworkers reported 1,4‐disila(Dewar‐benzene) (Me_3_Si)_4_C_4_Si_2_(Me)_2_ with the Si−Si σ bond [(2.246(2) Å] (Scheme 3).^[33]^ We found an elongation of the σ bond (Si⋅⋅⋅Si, 3.345 Å) for **1**, when coordinating the bulky groups L at Si and R^1^ and R^2^ at C. (Scheme 3). As a matter of fact, the Mulliken spin density distribution computations indicate that in **1** the electron spin density mainly locates at the four carbon atoms of the C_4_Si_2_ ring, and some reside at the adjacent alkynyl carbon atoms outside this ring. The spin density is C (bond to SiMe_3_) +0.19 and −0.19, C (bond to C≡CSiMe_3_) +0.16 and −0.16, and C (bond to CSiMe_3_ outside the ring) +0.12 and −0.12 respectively.^[23]^ The contribution from the two silicon atoms in the ring is almost negligible. This implies that the electron delocalization mode over the C_4_Si_2_ ring of **1** is elusive (Scheme 4), which is in sharp contrast to that for the C_6_ ring of benzene and the derivatives.^[15]^

[**1**]^2**+**^ shows the C−C bond lengths within the central C_4_Si_2_ ring close to those found in [**1**]^2+^ ⋅ 2[OS(O)_2_CF_3_]^−^ [1.356(6) Å],^[23]^ 1,4‐disila(Dewar‐benzene) [1.365(6) Å],^[33]^ as well as in [(L)_2_Si_2_C_4_(SiMe_3_)_2_(C_2_SiMe_3_)_2_](*μ*‐E) [E=O_2_, S, Se; 1.352(6)–1.377(3) Å],^[23]^ and **11** [1.367(2) Å], indicating that the double bond character is restricted within the cycle. Meanwhile, the Si−C bond lengths are close to those of the single bonds [1.868(2)–1.920(2) Å].[^22^, ^34^]

In comparison, [**1**]^+.^ displays the respective C−C and Si−C bond lengths in the C_4_Si_2_ ring intermediate between those in the rings of **1** and [**1**]^2**+**^. This is a consequence that one single electron is delocalized over the ring, as confirmed from the EPR and UV‐vis absorption data. However, the Mulliken spin density data suggest that this electron delocalization is dominantly over the two C_2_Si moieties of the C_4_Si_2_ ring.^[23]^ These electronic structures compare difference from those of the radical cations of benzene and the derivatives.^[15]^

Compound **11** displays a *μ*‐O_2_S(O)_2_ back coordination at the two Si atoms within the C_4_Si_2_ ring (Figure 5), showing a little different to the anion‐cation separated [**1**]^2+^ ⋅ 2[OS(O)_2_CF_3_]^−^.^[23]^ Such two Si atoms exhibit formally the positive charge although both are adopting the tetrahedral coordination sphere (Scheme 4). The Si−O_O2S(O)2_ bond lengths are 1.789(1) and 1.798(1) Å and a little longer than those of the Si−O_O2_ bonds in [(L)_2_Si_2_C_4_(SiMe_3_)_2_(C_2_SiMe_3_)_2_](*μ*‐O_2_) [1.745(1), 1.749(1) Å].^[23]^ It is worth noting that the formation of compounds **11** and [(L)_2_Si_2_C_4_(SiMe_3_)_2_(C_2_SiMe_3_)_2_](*μ*‐E) exhibits a pronounced region‐selective reactivity of **1** with respect to the two Si atoms within the C_4_Si_2_ ring.

The C_4_Si_2_ rings of **1**, [**1**]^+.^ in **2**–**8**, and [**1**]^2+^ in **9** and **10** show their planar arrangement with the least square planes (Δ) of 0.0078, 0.0019–0.0321, and 0.0078–0.0100 Å, respectively. The electron delocalization is occurring over both the C_4_Si_2_ rings of **1** and [**1**]^+.^ but showing essential differences to [**1**]^2+^. Furthermore, the C_4_Si_2_ ring planar feature changes due to the bridge coordination by the *μ*‐O_2_S(O)_2_ (in **11**) and *μ*‐E (in [**1**](*μ*‐E), E=O_2_, S, Se).^[23]^ In summary the N,N‐chelation of the L ligand at the Si atoms supports the C_4_Si_2_ ring planarity of [**1**]^2+^. The electron delocalization over the C_4_Si_2_ rings of **1** and [**1**]^+.^ plays an important role as well. The nucleus independent chemical shifts (NICS) at 1 Å above the C_4_Si_2_ rings were calculated to be 1.62 (**1**), −0.67 ([**1**]^+.^), and 1.40 ([**1**]^2+^), respectively.^[23]^

## Conclusion

In summary, we have investigated reactions using 1,4‐disilabenzene **1** with a series of organometallic and inorganic electron‐transferable oxidants, from which compounds **2**–**11** were produced and characterized. Compound **1** has been proved to be the open‐shell singlet diradical.^[23]^ Through one‐electron transfer, the radical cation [**1**]^+.^ was formed, pairing with the well‐separated anions as [*n*BuB(C_6_F_5_)_3_]^−^ (**2**), [B(C_6_F_5_)_4_]^−^ (**3**), [HB(C_6_F_5_)_3_]^−^ (**4**), [EtB(C_6_F_5_)_3_]^−^ (**5**), {[(C_6_F_5_)_3_Al]_2_(μ‐F)]^−^ (**6**), [*n*BuAl(C_6_F_5_)_3_]^−^ (**7**), and [Cu(C_6_F_5_)_2_]^−^ (**8**), respectively. By the two‐electron transfer, the dication [**1**]^2+^ was obtained with the isolated anions as [EtB(C_6_F_5_)_3_]^−^ (**9**) and [MeAl(C_6_F_5_)_3_]^−^ (**10**). Compound **11** is a neutral species showing back coordination of the [*μ*‐O_2_S(O)_2_]^2−^ to the two Si atoms of the central C_4_Si_2_ ring.

Both B(C_6_F_5_)_3_ and Al(C_6_F_5_)_3_ are the preferred one‐electron transferable oxidants.[^24^, ^25^, ^29^] They were able to abstract one electron from **1** to form [B(C_6_F_5_)_3_]^−.^ and [Al(C_6_F_5_)_3_]^−.^ that probably snatches the H atom from the solvent to produce [HB(C_6_F_5_)_3_]^−^ and [HAl(C_6_F_5_)_3_]^−^, respectively. Group exchange reactions of either [HB(C_6_F_5_)_3_]^−^ or [HAl(C_6_F_5_)_3_]^−^ might occur with the organometallic reagents as *n*BuLi, LiB(C_6_F_5_)_4_, LiHBEt_3_, (*o*‐RC_6_H_4_)AlH_2_, AlMe_3_, and AlEt_3_, respectively. Therefore, combinations as B(C_6_F_5_)_3_/*n*BuLi, B(C_6_F_5_)_3_/LiB(C_6_F_5_)_4_, B(C_6_F_5_)_3_/LiHBEt_3_, Al(C_6_F_5_)_3_/(*o*‐TMP‐C_6_H_4_)AlH_2_, B(C_6_F_5_)_3_/AlEt_3_, Al(C_6_F_5_)_3_/*n*BuLi, Al(C_6_F_5_)_3_/AlMe_3_ show the efficiency to produce the stable isolated target compounds. This chemistry is reported limitedly.^[29]^ The (CuC_6_F_5_)_4_ works well to oxidize **1** by forming Cu and [Cu(C_6_F_5_)_2_]^−^ along with [**1**]^2+^.

Both **1** and [**1**]^+.^ exhibit the electron delocalization over the central C_4_Si_2_ ring. However, [**1**]^2**+**^ loses this character due to the loss of two electrons. The planar geometry is present over the C_4_Si_2_ rings in **1**, [**1**]^+.^, and [**1**]^2+^.

## Experimental Section

**Materials and methods**: All manipulations were carried out under a dry argon or nitrogen atmosphere using Schlenk line and glovebox techniques. Solvents toluene and benzene were dried by refluxing with sodium/potassium benzophenone under N_2_ prior to use. Acetonitrile was dried over CaH_2_ under N_2_. The NMR (^1^H, ^13^C, ^19^F, ^29^Si) spectra were recorded on Bruker Avance II 400 or 500 MHz spectrometers. The melting point of the compound was measured in a sealed glass tube using the Büchi‐540 instrument. Elemental analysis was performed with a Thermo Quest Italia SPA EA 1110 instrument. EPR spectra of compounds **3**, **5**, and **8** were obtained using Bruker EMX plus‐6/1 and those of **2**, **4**, **6**, **7** with Bruker EMX Plus‐10/12 both equipped with X‐band variable‐temperature apparatus. UV‐vis spectra were recorded on Lambda 750 spectrometer. Commercial reagents were purchased from Aldrich, Acros, or Alfa‐Aesar Chemical Co. and used as received. Compounds (L)_2_Si_2_C_4_(SiMe_3_)_2_(C_2_SiMe_3_)_2_ (**1**, L=PhC(N*t*Bu)_2_]),^[23]^ (*o*‐RC_6_H_4_)AlH_2_ (R=N(CMe_2_CH_2_)_2_CH_2_),^[28]^ LiB(C_6_F_5_)_4_,^[35]^ B(C_6_F_5_)_3_,^[36]^ Al(C_6_F_5_)_3_,^[37]^ and (CuC_6_F_5_)_4_ ^[38]^ were prepared according to literature procedures. Deposition Number(s) 2103401 (for **2**), 2103402 (for **4** ⋅ C_6_H_6_), 2103403 (for **5** ⋅ 0.2 C_6_H_14_), 2103404 (for **6**), 2103405 (for **7**), 2103406 (for **8**), 2103407 (for **9** ⋅ C_6_H_6_), 2103408 (for **10** ⋅ 2.5 C_6_H_6_), 2103409 (for **11**) contain(s) the supplementary crystallographic data for this paper. These data are provided free of charge by the joint Cambridge Crystallographic Data Centre and Fachinformationszentrum Karlsruhe Access Structures service.

**Synthesis of [1]^+.^[*n*BuB(C_6_F_5_)_3_]^−^ (2)**: At room temperature to a mixture of **1** (0.027 g, 0.03 mmol) and *n*BuLi (2.4 M in *n*‐hexane, 12.4 μL, 0.03 mmol) in C_6_H_6_ (0.7 mL) was added B(C_6_F_5_)_3_ (0.015 g, 0.03 mmol). A deep brown solution was quickly formed. The mixture solution was stored at room temperature for 12 h, giving yellow block crystals of **2**. These crystals were collected by filtration. Yield: 0.022 g, 50 %. Mp: 198 °C (dec.). Anal. calcd. (%) for C_72_H_91_BF_15_N_4_Si_6_ (*M*_r_=1476.84): C, 58.56; H, 6.21; N, 3.79. Found: C, 58.53; H, 6.19; N, 3.82.

**Synthesis of [1]^+.^[B(C_6_F_5_)_4_]^−^ (3)**: At room temperature to a mixture of **1** (0.027 g, 0.03 mmol) and LiB(C_6_F_5_)_4_ (0.015 g, 0.03 mmol) in C_6_H_6_ (0.7 mL) was added B(C_6_F_5_)_3_ (0.015 g, 0.03 mmol). A deep brown solution was quickly formed. The mixture solution was stored at room temperature for 12 h, giving yellow block crystals of **3**. These crystals were collected by filtration. Yield: 0.022 g, 50 %. Mp: 198 °C (dec.). Anal. calcd. (%) for C_72_H_91_BF_15_N_4_Si_6_ (*M*_r_=1476.84): C, 58.56; H, 6.21; N, 3.79. Found: C, 58.53; H, 6.19; N, 3.82.

**Synthesis of [1]^+.^[HB(C_6_F_5_)_3_]^−^ (4)**: Method A: At room temperature to a mixture of **1** (0.027 g, 0.03 mmol) and LiHBEt_3_ (1 M in THF, 30 μL, 0.03 mmol) in C_6_H_6_ (0.7 mL) was added B(C_6_F_5_)_3_ (0.015 g, 0.03 mmol). After addition, a deep brown solution was quickly formed. The mixture solution has stored at room temperature for 12 h, giving green block crystals of **4**. These crystals were collected by filtration. Yield: 0.020 g, 47 %. Mp: 165 °C (dec.). Anal. calcd. (%) for C_68_H_83_BF_15_N_4_Si_6_ (*M*_r_=1420.74): C, 57.49; H, 5.89; N, 3.94. Found: C, 57.45; H, 5.90; N, 3.98. Method B: At room temperature to a mixture of **1** (0.027 g, 0.03 mmol) and (*o*‐RC_6_H_4_)AlH_2_ (0.007 g, 0.03 mmol) in C_6_H_6_ (0.7 mL) was added B(C_6_F_5_)_3_ (0.015 g, 0.03 mmol). After addition, a deep brown solution was quickly formed. The mixture solution was stored at room temperature for 12 h, giving green block crystals of **4**. These crystals were collected by filtration. Yield: 0.026 g, 60 %. Preliminary X‐ray diffraction measurement determined the unit cell parameters that are the same as those for compound obtained in method A.

**Synthesis of [1]^+.^[EtB(C_6_F_5_)_3_]^−^ (5)**: At room temperature to a mixture of B(C_6_F_5_)_3_ (0.015 g, 0.03 mmol) and AlEt_3_ (1.0 M in *n*‐hexane, 30 μL, 0.03 mmol) in C_6_H_6_ (0.7 mL) was added **1** (0.027 g, 0.03 mmol). After addition, a deep brown solution was quickly formed. The mixture solution was kept at room temperature for 12 h, giving yellow block crystals of **5**. These crystals were collected by filtration. Yield: 0.018 g, 41 %. Mp: 192 °C (dec.). Anal. calcd. (%) for C_70_H_87_BF_15_N_4_Si_6_ (*M*_r_=1448.79): C, 58.03; H, 6.05; N, 3.87. Found: C, 58.01; H, 6.02; N, 3.89.

**Synthesis of [1]^+.^{[(C_6_F_5_)_3_Al]_2_(*μ*‐F)}^−^ (6)**: At room temperature to a mixture of Al(C_6_F_5_)_3_ (0.036 g, 0.06 mmol) and **1** (0.054 g, 0.06 mmol) was added C_6_H_6_ (1.0 mL). After addition, a deep brown solution was quickly formed. The mixture solution was stored at room temperature for 12 h, giving yellow block crystals of **6**. These crystals were collected by filtration. Yield: 0.018 g, 30 %. Mp: 231 °C (dec.). Anal. calcd. (%) for C_86_H_82_Al_2_F_31_N_4_Si_6_ (*M*_r_=1983.05): C, 52.09; H, 4.17; N, 2.83. Found: C, 52.06; H, 4.14; N, 2.85.

**Synthesis of [1]^+.^[*n*BuAl(C_6_F_5_)_3_]^−^ (7)**: At room temperature to a mixture of **1** (0.027 g, 0.03 mmol) and *n*BuLi (2.4 M in *n*‐hexane, 12.4 μL, 0.03 mmol) in C_6_H_6_ (0.7 mL) was added Al(C_6_F_5_)_3_ (0.018 g, 0.03 mmol). After addition, a deep brown solution was quickly formed. The mixture solution was stored at room temperature for 12 h, giving yellow block crystals of **7**. These crystals were collected by filtration. Yield: 0.030 g, 67 %. Mp: 194 °C (dec.). Anal. calcd. (%) for C_72_H_91_AlF_15_N_4_Si_6_ (*M*_r_=1493.02): C, 57.92; H, 6.14; N, 3.75. Found: C, 57.88; H, 6.10; N, 3.77.

**Synthesis of [1]^+.^[Cu(C_6_F_5_)_2_]^−^ (8)**: At room temperature to a mixture of (CuC_6_F_5_)_4_ (0.014 g, 0.015 mmol) and **1** (0.027 g, 0.03 mmol) was added C_6_H_6_ (0.5 mL). After addition, a deep brown solution was quickly formed together with the Cu mirror. By filtration to remove the Cu mirror, the mixture was solution kept at room temperature for 12 h, giving yellow‐green block crystals of **8**. These crystals were collected by filtration. Yield for **8**: 0.028 g, 54 %. Mp: 186 °C (dec.). Anal. calcd (%) for C_74_H_82_Cu_2_F_21_N_4_Si_6_ (*M*_r_=1722.07): C, 51.61; H, 4.80; N, 3.25. Found: C, 51.58; H, 4.79; N, 3.29.

**Synthesis of [1]^2+^** **⋅ 2[EtB(C_6_F_5_)_3_]^−^ (9)**: At room temperature to a mixture of B(C_6_F_5_)_3_ (0.051 g, 0.1 mmol) and AlEt_3_ (1 M in THF, 0.1 mL, 0.1 mmol) in CH_3_CN (10 mL) was added **1** (0.045 g, 0.05 mmol). After addition, the solution color changed into deep brown. Further color changes to yellow, light yellow, and then almost colorless were observed. The mixture solution was stored at −20 °C for 24 h, giving colorless block crystals of **9**. Yield: 0.16 g, 20 %. Mp: 216 °C (dec.). ^1^H NMR (500 MHz, CD_3_CN, 298 K, ppm): *δ*=0.85 (br, 6 H, CH_2_C*H*_3_), 0.95 (s, 18 H, Si*Me*_3_), 1.21 (s, 18 H, Si*Me*_3_), 1.50 (s, 36 H, *tBu*), 1.95 (br, 4 H, C*H*_2_CH_3_), 7.21–7.83 (m, 10 H, *Ph*). ^19^F NMR (376 MHz, CD_3_CN, 298 K, ppm): *δ*=−134.49 (*o*‐*F*, 12 F), −158.06 (*p*‐*F*, 6 F), −165.13 (*m*‐*F*, 12 F). The ^13^C, ^11^B, and ^29^Si NMR data were not obtained due to the not good solubility. Anal. calcd. (%) for C_90_H_92_B_2_F_30_N_4_Si_6_ (*M*_r_=1989.84): C, 54.33; H, 4.66; N, 2.82. Found: C, 54.31; H, 4.68; N, 2.82.

**Synthesis of [1]^2+^** **⋅ 2[MeAl(C_6_F_5_)_3_]^−^ (10)**: At room temperature to a mixture of Al(C_6_F_5_)_3_ (0.032 g, 0.06 mmol) and AlMe_3_ (1.0 M in *n*‐hexane, 60 μL, 0.06 mmol) in CH_3_CN (1.0 mL) was added **1** (0.027 g, 0.03 mmol). After addition, the solution color changed into deep brown. Further color changes to yellow, light yellow, and then almost colorless were observed. The mixture solution was stored at −20 °C for 24 h, giving colorless block crystals of **10**. The crystals were collected by filtration. Yield: 0.020 g, 33 %. Mp: 198 °C (dec.). ^1^H NMR (500 MHz, CD_3_CN, 298 K, ppm): *δ*=−0.46 (s, 6 H, Al*Me*), 1.22 (s, 18 H, Si*Me*_3_), 1.27 (s, 18 H, Si*Me*_3_), 2.32 (s, 36 H, *tBu*), 6.95–7.54 (m, 10 H, Ph). ^19^F NMR (376 MHz, CD_3_CN, 298 K, ppm): *δ*=−123.53 (*o*‐*F*, 12 F), −153.25 (*p*‐*F*, 6 F), and −162.42 (*m*‐*F*, 12 F). The ^13^C, ^27^Al, and ^29^Si NMR data were not obtained due to the not good solubility. Anal. calcd. (%) for C_88_H_88_Al_2_F_30_N_4_Si_6_ (*M*_r_=1994.13): C, 53.00; H, 4.45; N, 2.81. Found: C, 52.96; H, 4.42; N, 2.85.

**Synthesis of [(L)_2_Si_2_C_4_(SiMe_3_)_2_(C_2_SiMe_3_)_2_][*μ*‐O_2_S(O)_2_] (11)**: At room temperature to a mixture of Ag_2_SO_4_ (0.012 g, 0.04 mmol) and **1** (0.036 g, 0.04 mmol) was added toluene (10 mL). After addition, the mixture was stirred for 2 h to give a colorless solution along with a black silver mirror. The silver mirror was removed by filtration. The resulting solution was stored at −20 °C for 24 h, giving colorless block crystals of **11**. The crystals were collected by filtration. Yield: 0.032 g, 80 %. Mp: 209 °C (dec.). ^1^H NMR (600 MHz, C_6_D_6_, 298 K, ppm): *δ*=0.35 (s, 18 H, Si*Me*_3_), 0.77 (s, 18 H, Si*Me*_3_), 1.05 (s, 18 H, C*Me*_3_), 1.45 (s, 18 H, C*Me*_3_), 6.97–7.84 (m, 10 H, *Ph*). ^13^C NMR (151 MHz, C_6_D_6_, 298 K, ppm): *δ*=0.12 (Si*Me*_3_), 1.15 (Si*Me*_3_), 33.09 (C*Me*_3_), 33.41 (C*Me*_3_), 55.24 (*C*Me_3_), 57.61 (*C*Me_3_), 104.27 (≡*C*SiMe_3_), 110.54 (≡*C*C), 127.49, 127.63, 128.35, 129.07, 129.13, 130.06, 134.84 (*Ph*), 150.91 (=*C*C), 173.21 (=*C*SiMe_3_), 173.43 (N*C*N). ^29^Si NMR (79 MHz, C_6_D_6_, 298 K, ppm): *δ*=−105.05 (N*Si*N), −19.43 (*Si*Me_3_), −6.95 (*Si*Me_3_). Anal. calcd. (%) for C_50_H_82_Si_6_N_4_SO_4_ (*M*_r_=1003.80): C, 59.83; H, 8.23; N, 5.58. Found: C, 59.81; H, 8.22; N, 5.60.

## Conflict of interest

The authors declare no conflict of interest.

1

## Supporting information

As a service to our authors and readers, this journal provides supporting information supplied by the authors. Such materials are peer reviewed and may be re‐organized for online delivery, but are not copy‐edited or typeset. Technical support issues arising from supporting information (other than missing files) should be addressed to the authors.

Supporting Information

## Data Availability

The data that support the findings of this study are available on request from the corresponding author. The data are not publicly available due to privacy or ethical restrictions.
