# Supplementary material for: One‐ and Two‐Electron Transfer Oxidation of 1,4‐Disilabenzene with Formation of Stable Radical Cations and Dications
Source: Chemistry. 2021 Dec 16;28(5):e202103715. doi: 10.1002/chem.202103715 (PMC9299862; doi:10.1002/chem.202103715)
Supplement: Supplementary file 1 — Supporting Information [file CHEM-28-0-s001.pdf]

Chemistry–A European Journal

Supporting Information

One- and Two-Electron Transfer Oxidation of 1,4-Disilabenzene with Formation of Stable Radical Cations and Dications

Yilin Chen, Zhikang Chen, Liuyin Jiang,* Jiancheng Li, Yiling Zhao, Hongping Zhu,* and Herbert W. Roesky*

Content:

I. X-Ray Crystallographic Analysis

II. Selected crystal structures and bond parameters

III. Selected EPR spectra

IV. Selected UV-vis absorption spectra

V. Collected ^{1}H, ^{13}C, ^{19}F, and ^{29}Si NMR spectra of compounds 9−11

I. X-Ray Crystallographic Analysis

Crystallographic data of **2**, **4** C_6H_6, **5** $0.2C_6H_{14}$, **6–8**, **9** C_6H_6, **10** $2.5C_6H_6$, and 11 were all collected on XtaLAB Synergy, Dualflex, HyPix diffractometer (Cu-Kα radiation, λ = 1.54184 Å). Absorption corrections were applied by using the spherical harmonics program (multi-scan type). All structures were solved by direct methods (SHELXS-2015)[S1] and refined against F^2 using SHELXL-2017/1.[S2] In general, the non-hydrogen atoms were located by difference Fourier synthesis and refined anisotropically, and hydrogen atoms were included using a riding model with U_{iso} tied to the U_{iso} of the parent atoms unless otherwise specified. In **2**, two methyl groups from the tBu of one amidinato ligand were disordered and treated by the PART method, and final refinements gave C(21) and C(21A) with the respective occurancies of 0.80595 and 0.19405 and C(22) and C(22A) of the same respective occurancies. Three methyl groups from the tBu of another amidinato ligand were disordered and treated by the PART method, and final refinements gave C(36) and C(36A) with the respective occupancies of 0.61103 and 0.38897, so it was with C(37) and C(37A) and C(38) and C(38A). One phenyl group from the amidinato ligand were also disordered and treated by the PART method, and final refinements gave C(29)C(30)C(31)C(32)C(33)C(34) and C(29A)C(30A)C(31A) C(32A)C(33A)C(34A) with the respective occurancies of 0.41514 and 0.58486. Three methyl groups from the $SiMe_3$ group were disordered as well and treated by the PART method, and final refinements gave C(42) and C(42A) with the respective occurancies of 0.35701 and 0.64299, so it was with C(43) and C(43A) and C(44) and C(44A). Finally, the nBu group from the anion $[nBuB(C_6F_5)_3]^-$ was disordered and treated by the PART method, and final refinements gave C(51)C(52)C(53)C(54) and C(51A)C(52A)C(53A)C(54A) with the respective occurancies of 0.50979 and 0.49021. In **4** C_6H_6, two half moieties of the cationic part were disclosed and then the two whole cations were obtained by symmetric operation. The H atom of the anion $[HB(C_6F_5)_3]^-$ was located by difference Fourier synthesis and refined isotropically in an AFIX 13 type. A half moiety of the C_6H_6 was disclosed, which was disordered and treated by the PART method, and final refinements gave C(69)C(70)C(71) and C(69A)C(70A)C(71A) with the respective occurancies of 0.61092 and 0.38908. In **5** $0.2C_6H_{14}$, two half moieties of the cationic part were disclosed and then the two whole cations were obtained by symmetric operation. In one half moiety of the cation, one phenyl group from the amidinato ligand were disordered and treated by the PART method, and final refinements gave C(10)C(11)C(12)C(13)C(14)C(15) and C(10A)C(11A)C(12A)C(13A)C(14A)C(15A) with the respective occurancies of 0.55022 and 0.44978. Three methyl groups from the $SiMe_3$ group were disordered and treated by the PART method, and final refinements gave C(23) and C(23A) with the respective occurancies of 0.59046 and 0.40954, so it was with C(24) and C(24A) and C(25) and C(25A). In another half moiety of the cation, three methyl groups from the $SiMe_3$ group were disordered and treated by the PART method, and final refinements gave C(48) and C(48A) with the respective occurancies of 0.55874 and 0.45126, so it was with C(49) and C(49A) and C(50) and C(50A). The *n*-hexane molecule C(71)C(72)C(73)C(74)C(75)C(76) was disclosed with occurancy better being as 0.2. Due to a serious disordering of this molecule, the six carbon atoms were refined isotropically where the hydrogen atoms were not able to be added geomertically. In **6**, a half moiety of the cationic part was disclosed and then the whole cation was obtained by symmetric operation, so it was with the half moiety of the anionic part. In **7**, two half moieties of the cationic part were disclosed and then the two whole cations were obtained by symmetric operation. In one half moiety of the cation, three methyl groups from the $SiMe_3$ group were disordered and treated by the PART method, and final refinements gave C(23) and C(23A) with the respective occurancies of 0.55489 and 0.44511, so it was with C(24) and C(24A) and C(25) and C(25A). In 8, a half moiety of the cationic part was disclosed and then the whole cation was obtained by symmetric operation, so it was with the half moiety of the anionic part. In **9** C_6H_6, a half

moiety of the cationic part was disclosed and then the whole cation was obtained by symmetric operation. In **10**·2.5C_6H_6, a half moiety of the cationic part was disclosed and then the whole cation was obtained by symmetric operation. In this cationic part, one methyl group from the $SiMe_3$ group was disordered and treated by the PART method, and final refinements gave C(8) and C(8A) with the respective occurancies of 0.45269 and 0.54731. A half C_6H_6 molecule was disclosed and refined with occurany of 0.5. In **11**, two oxygen atoms from the SO_4 group were disclosed and treated by the PART method, and final refinements gave O(3) and O(3A) with the respective occurancies of 0.72828 and 0.27172, so it was with O(4) and O(4A). Three methyl groups from the $SiMe_3$ group were disordered and treated by the PART method, and final refinements gave C(42) and C(42A) with the respective occurancies of 0.51449 and 0.48551, so it was with C(43) and C(43A) and C(44) and C(44A). A summary of cell parameters, data collection, and structure solution and refinements is given in Table S1.

References

(S1) G. M. Sheldrick, Crystal structure refinement with SHELXL. *Acta Cryst. Sect. C*, 2015, **71**, 3–8.
(S2) G. M. Sheldrick, A short history of SHELX. *Acta Cryst. Sect. A*, 2008, **64**, 112–122.

Table S1. Crystal data and structure refinements for complexes **2**, **4** $\cdot C_6H_6$, **5** $\cdot 0.2C_6H_{14}$, **6–8**, **9** $\cdot C_6H_6$, **10** $\cdot 2.5C_6H_6$, and **11**

	**2**	**4** $\cdot C_6H_6$	**5** $\cdot 0.2C_6H_{14}$
CCDC	2103401	2103402	2103403
formula	$C_{72}H_{91}BF_{15}N_4Si_6$	$C_{71}H_{86}BF_{15}N_4Si_6$	$C_{71.2}H_{87}BF_{15}N_4Si_6$
fw	1476.83	1459.78	1463.19
cryst syst	Monoclinic	Triclinic	Triclinic
space group	$P2_1/c$	P–1	P–1
a/Å	11.35070(10)	13.0955(3)	13.16220(10)
b/Å	39.0397(6)	14.1191(3)	14.8559(2)
c/Å	17.5774(3)	21.6165(3)	21.2450(2)
α/deg	90	94.658(2)	91.6830(10)
β/deg	92.9660(10)	98.998(2)	100.1040(10)
γ/deg	90	106.293(2)	106.1950(10)
V/Å^3	7778.60(19)	3755.97(14)	3913.96(7)
Z	4	2	2
ρ_{calcd}/g·cm^{-3}	1.261	1.291	1.242
μ/mm^{-1}	1.674	1.729	1.660
F(000)	3100	1528	1532
crystal size/mm^3	0.40×0.10×0.05	0.40×0.20×0.05	0.40×0.20×0.05
θ range/deg	2.76–75.38	3.29–67.58	3.11–74.55
index ranges	$-13 \le h \le 14$<br>$-48 \le k \le 32$<br>$-21 \le l \le 22$	$-15 \le h \le 15$<br>$-16 \le k \le 16$<br>$-25 \le l \le 25$	$-16 \le h \le 16$<br>$-18 \le k \le 18$<br>$-26 \le l \le 26$
collected data	50447	42718	48232
unique data	15437<br>(R_{int} = 0.0574)	13137<br>(R_{int} = 0.0336)	15537<br>(R_{int} = 0.0392)
completeness to θ (%)	96.0	96.9	97.0
data/restraints/params	15437/814/1060	13137/96/926	15537/612/1007
GOF on F^2	1.054	1.027	1.045
final R indices [I > 2(I)]	R_1 = 0.0818<br>wR_2 = 0.1882	R_1 = 0.0453<br>wR_2 = 0.1087	R_1 = 0.0460<br>wR_2 = 0.1224
R indices (all data)	R_1 = 0.1108<br>wR_2 = 0.2053	R_1 = 0.0539<br>wR_2 = 0.1134	R_1 = 0.0488<br>wR_2 = 0.1246
Largest diff peak/hole (e·Å^{-3})	0.64/–0.51	0.78/–0.70	0.66/–0.70

[a] $R_1 = \sum(||F_o|-|F_c||)/\sum|F_o|$, $wR_2 = [\sum w(F_o^2-F_c^2)^2/\sum w(F_o^2)]^{1/2}$, GOF = $[\sum w(F_o^2-F_c^2)^2/(N_o-N_p)]^{1/2}$.

(to be continued)

	**6**	**7**	**8**
CCDC	2103404	2103405	2103406
formula	$C_{86}H_{82}Al_2F_{31}N_4Si_6$	$C_{72}H_{91}AlF_{15}N_4Si_6$	$C_{62}H_{82}CuF_{10}N_4Si_6$
fw	1983.05	1493.00	1305.39
cryst syst	Triclinic	Triclinic	Triclinic
space group	*P*–1	*P*–1	*P*–1
a/Å	11.1292(3)	11.44880(10)	11.4029(5)
b/Å	11.6914(3)	13.1172(2)	11.9486(7)
c/Å	18.8610(5)	27.1344(3)	14.1016(8)
α/deg	89.878(2)	77.3570(10)	66.681(5)
β/deg	73.327(2)	84.1070(10)	84.748(4)
γ/deg	75.653(2)	84.4010(10)	74.083(4)
V/Å^3	2271.32(11)	3943.27(8)	1696.42(17)
Z	1	2	1
ρ_{calcd}/g·cm^{-3}	1.450	1.257	1.278
μ/mm^{-1}	2.033	1.762	2.039
F(000)	1015	1566	685
crystal size/mm^3	0.40×0.10×0.05	0.40×0.10×0.05	0.30×0.20×0.02
θ range/deg	3.91–75.17	3.35–75.34	3.41–74.57
index ranges	$-13 \le h \le 12$<br>$-13 \le k \le 14$<br>$-23 \le l \le 23$	$-14 \le h \le 11$<br>$-16 \le k \le 15$<br>$-33 \le l \le 34$	$-14 \le h \le 12$<br>$-14 \le k \le 14$<br>$-15 \le l \le 17$
collected data	24952	51458	18973
unique data	9096<br>(R_{int} = 0.0491)	15906<br>(R_{int} = 0.0477)	6620<br>(R_{int} = 0.0641)
completeness to θ (%)	97.0	97.3	95.4
data/restraints/params	9096/0/596	15906/131/939	6620/0/388
GOF on F^2	1.029	1.034	1.059
final R indices [$I > 2(I)$]	R_1 = 0.0511<br>wR_2 = 0.1416	R_1 = 0.0582<br>wR_2 = 0.1541	R_1 = 0.0592<br>wR_2 = 0.1450
R indices (all data)	R_1 = 0.0543<br>wR_2 = 0.1445	R_1 = 0.0647<br>wR_2 = 0.1589	R_1 = 0.0745<br>wR_2 = 0.1524
Largest diff peak/hole (e·Å^{-3})	0.55/–0.54	0.64/–0.53	1.65/–0.58

^{a} $R_1 = \sum(||F_o|-|F_c||)/\sum|F_o|$, $wR_2 = [\sum w(F_o^2-F_c^2)^2/\sum w(F_o^2)]^{1/2}$, GOF = $[\sum w(F_o^2-F_c^2)^2/(N_o-N_p)]^{1/2}$.

(to be continued)

	**9** C_6H_6	**10** ·2.5C_6H_6	**11**
CCDC	2103407	2103408	2103409
formula	$C_{102}H_{104}B_2F_{30}N_4Si_6$	$C_{103}H_{103}Al_2F_{30}N_4Si_6$	$C_{50}H_{82}SO_4N_4Si_6$
fw	2146.05	2189.39	1003.79
cryst syst	Triclinic	Triclinic	Triclinic
space group	*P*–1	*P*–1	*P*–1
a/Å	14.2307(4)	14.5978(5)	10.3621(2)
b/Å	14.7041(4)	14.8593(5)	13.0587(3)
c/Å	14.7163(4)	14.9422(6)	21.4315(4)
α/deg	113.443(3)	63.596(3)	89.607(2)
β/deg	102.102(2)	66.053(3)	81.034(2)
γ/deg	105.600(2)	77.842(3)	89.210(2)
V/Å^3	2540.43(14)	2651.36(19)	2864.28(10)
Z	1	1	2
ρ_{calcd}/g·cm^{-3}	1.403	1.371	1.164
μ/mm^{-1}	1.684	1.781	2.042
F(000)	1108	1129	1084
crystal size/mm^3	0.40×0.20×0.10	0.60×0.40×0.40	0.30×0.20×0.10
θ range/deg	3.45–71.68	3.32–73.99	3.39–65.88
index ranges	$-17 \le h \le 17$<br>$-17 \le k \le 11$<br>$-17 \le l \le 18$	$-17 \le h \le 18$<br>$-18 \le k \le 17$<br>$-18 \le l \le 16$	$-12 \le h \le 12$<br>$-15 \le k \le 15$<br>$-25 \le l \le 21$
collected data	30292	31999	31563
unique data	9452<br>(R_{int} = 0.0333)	10521<br>(R_{int} = 0.0316)	9698<br>(R_{int} = 0.0271)
completeness to θ (%)	95.0	95.6	97.3
data/restraints/params	9452/0/662	10521/23/691	9698/126/660
GOF on F^2	1.058	1.087	1.032
final R indices [I > 2(I)]	R_1 = 0.0348<br>wR_2 = 0.0890	R_1 = 0.0612<br>wR_2 = 0.1563	R_1 = 0.0308<br>wR_2 = 0.0809
R indices (all data)	R_1 = 0.0388<br>wR_2 = 0.0920	R_1 = 0.0664<br>wR_2 = 0.1600	R_1 = 0.0334<br>wR_2 = 0.0824
Largest diff peak/hole (e·Å^{-3})	0.29/–0.30	0.83/–1.03	0.35/–0.26

[a] $R_1 = \sum(||F_o|-|F_c||)/\sum|F_o|$, $wR_2 = [\sum w(F_o^2-F_c^2)^2/\sum w(F_o^2)]^{1/2}$, GOF = $[\sum w(F_o^2-F_c^2)^2/(N_o-N_p)]^{1/2}$.

II. Selected crystal structures and bond parameters

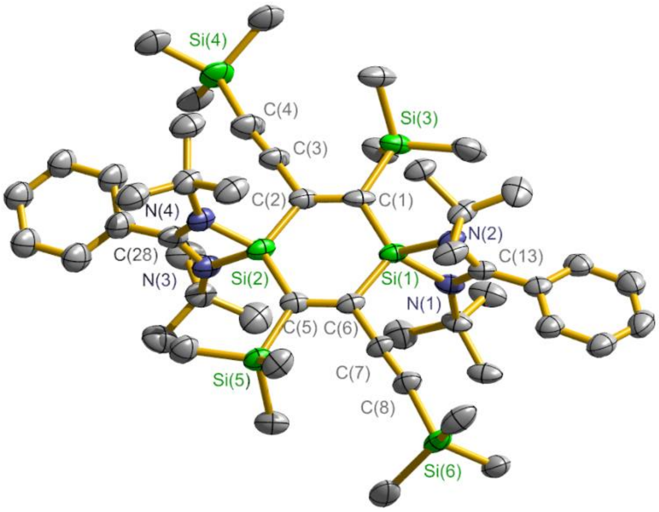

Figure S1. X-ray crystal structure of $[\mathbf{1}]^{+\cdot}$ in **2** with thermal ellipsoids at 50% probability level. The hydrogen atoms are omitted for clarity. Selected bond lengths (Å) and angles (°): Si(1)–C(1) 1.813(4), Si(1)–C(6) 1.838(4), Si(2)–C(2) 1.833(4), Si(2)–C(5) 1.806(4), C(1)–Si(3) 1.892(4), C(1)–C(2) 1.396(6), C(2)–C(3) 1.432(5), C(3)–C(4) 1.202(5), C(4)–Si(4) 1.853(4), C(5)–Si(5) 1.887(4), C(5)–C(6) 1.405(5), C(6)–C(7) 1.426(4), C(7)–C(8) 1.214(5), C(8)–Si(6) 1.844(4), Si(1)–N(1) 1.806(3), Si(1)–N(2) 1.806(3), C(13)–N(1) 1.346(4), C(13)–N(2) 1.339(4), Si(2)–N(3) 1.810(3), Si(2)–N(4) 1.806(3), C(28)–N(3) 1.335(5), C(28)–N(4) 1.339(5); C(1)–Si(1)–C(6) 115.63(18), Si(1)–C(6)–C(5) 125.9(2), C(6)–C(5)–Si(2) 117.9(3), C(5)–Si(2)–C(2) 116.32(18), Si(2)–C(2)–C(1) 125.6(3), C(2)–C(1)–Si(1) 118.5(3), Si(1)–C(1)–Si(3) 122.3(2), C(1)–C(2)–C(3) 120.9(4), C(2)–C(3)–C(4) 176.9(5), C(3)–C(4)–Si(4) 171.2(5), Si(2)–C(5)–Si(5) 122.7(2), C(5)–C(6)–C(7) 121.4(3), C(6)–C(7)–C(8) 176.5(4), C(7)–C(8)–Si(6) 169.7(4), N(1)–Si(1)–N(2) 72.20(13), N(1)–C(13)–N(2) 104.8(3), N(3)–Si(2)–N(4) 72.48(15), N(3)–C(28)–N(4) 106.2(3).

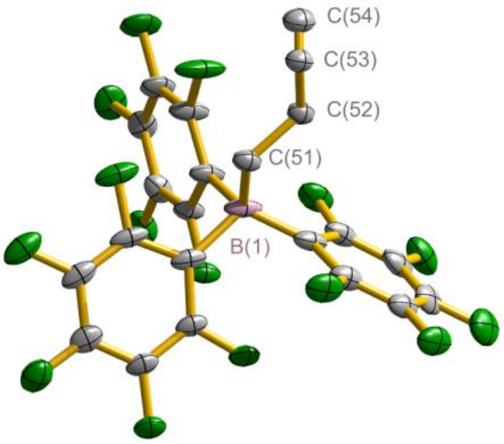

Figure S2. X-ray crystal structure of $[n\text{BuB}(C_6F_5)_3]^-$ in **2** with thermal ellipsoids at 50% probability level. The hydrogen atoms are omitted for clarity.

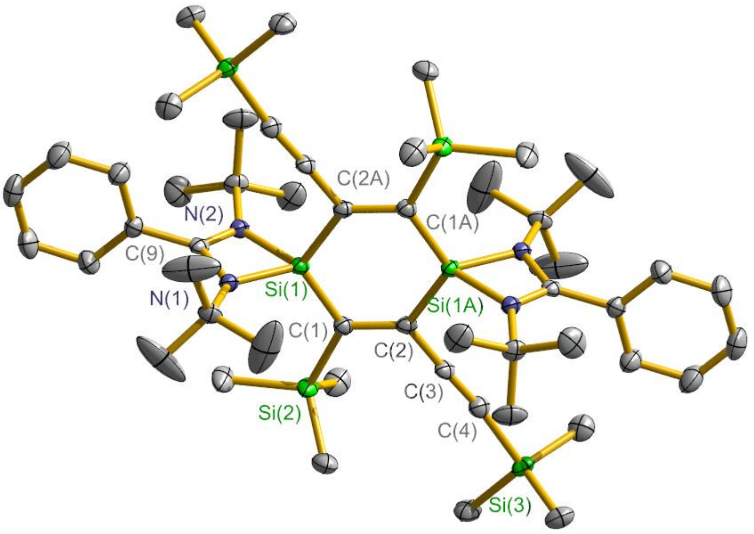

Figure S3. X-ray crystal structure of [**1**]$^{+\cdot}$ in **4** with thermal ellipsoids at 50% probability level. The hydrogen atoms are omitted for clarity. Selected bond lengths (Å) and angles (°): Si(1)–C(1) 1.805(2), Si(1)–C(2A) 1.847(2), C(1)–C(2) 1.401(3), C(2)–C(3) 1.421(3), C(3)–C(4) 1.217(3), C(4)–Si(3) 1.840(2), Si(1)–N(1) 1.8111(18), Si(1)–N(2) 1.8123(18), C(9)–N(1) 1.341(3), C(9)–N(2) 1.338(3); C(1)–Si(1)–C(2A) 116.32(10), Si(1)–C(1)–C(2) 118.08(15), C(1)–C(2)–Si(1A) 125.60(16), C(1)–C(2)–C(3) 122.02(18), C(2)–C(3)–C(4) 177.2(2), C(3)–C(4)–Si(3) 172.2(2), N(1)–Si(1)–N(2) 72.25(8), N(1)–C(9)–N(2) 105.75(18).

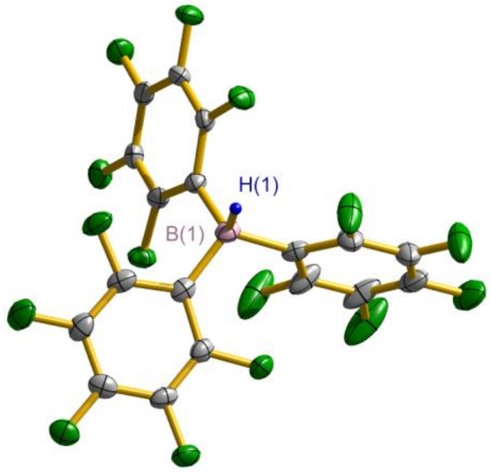

Figure S4. X-ray crystal structure of $[HB(C_6F_5)_3]^-$ in **4** with thermal ellipsoids at 50% probability level.

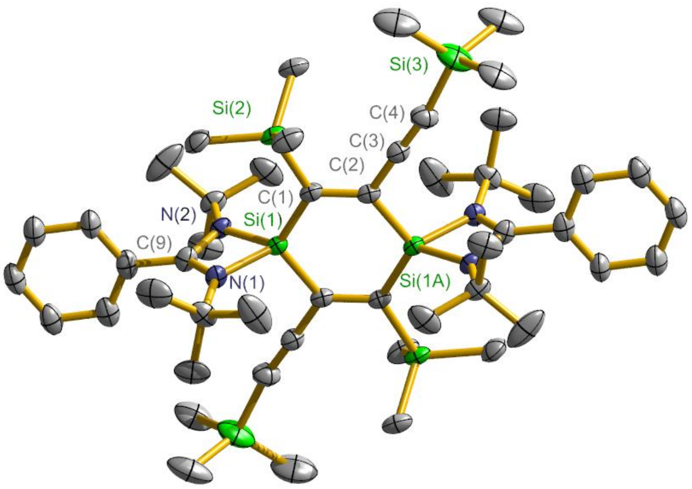

Figure S5. X-ray crystal structure of $[\mathbf{1}]^{+\cdot}$ in **5** with thermal ellipsoids at 50% probability level. The hydrogen atoms are omitted for clarity. Selected bond lengths (Å) and angles (°): Si(1)–C(1) 1.8057(16), Si(1)–C(2A) 1.8392(16), C(1)–C(2) 1.404(2), C(2)–C(3) 1.429(2), C(3)–C(4) 1.205(3), C(4)–Si(3) 1.8369(19), Si(1)–N(1) 1.8033(14), Si(1)–N(2) 1.8121(14), C(9)–N(1) 1.341(2), C(9)–N(2) 1.340(2); C(1)–Si(1)–C(2A) 115.76(7), Si(1)–C(1)–C(2) 118.47(11), C(1)–C(2)–Si(1A) 125.77(12), C(1)–C(2)–C(3) 121.24(14), C(2)–C(3)–C(4) 178.17(19), C(3)–C(4)–Si(3) 171.40(18), N(1)–Si(1)–N(2) 72.58(6), N(1)–C(9)–N(2) 105.91(14).

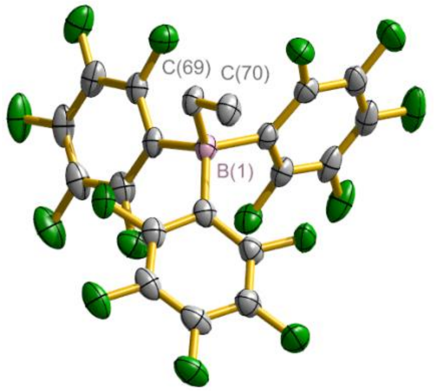

Figure S6. X-ray crystal structure of $[EtB(C_6F_5)_3]^-$ in **5** with thermal ellipsoids at 50% probability level. The hydrogen atoms are omitted for clarity.

Selected bond lengths (Å) and angles (°) for $[\mathbf{1}]^{+\cdot}$ in **6**: Si(1)–C(1) 1.8094(19), Si(1)–C(2A) 1.8423(18), C(1)–C(2) 1.406(2), C(2)–C(3) 1.428(3), C(3)–C(4) 1.221(3), C(4)–Si(3) 1.841(2), Si(1)–N(1) 1.8147(16), Si(1)–N(2) 1.8149(16), C(9)–N(1) 1.335(2), C(9)–N(2) 1.341(2); C(1)–Si(1)–C(2A) 115.75(8), Si(1)–C(1)–C(2) 118.96(13), C(1)–C(2)–Si(1A) 125.13(14), C(1)–C(2)–C(3) 121.74(16), C(2)–C(3)–C(4) 178.8(2), C(3)–C(4)–Si(3) 170.63(18), N(1)–Si(1)–N(2) 72.30(7), N(1)–C(9)–N(2) 106.31(16).

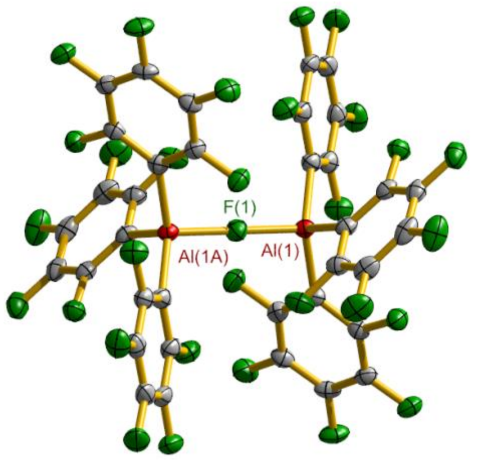

Figure S7. X-ray crystal structure of $\{[Al(C_6F_5)_3](\mu\text{-}F)\}^-$ in **6** with thermal ellipsoids at 50% probability level.

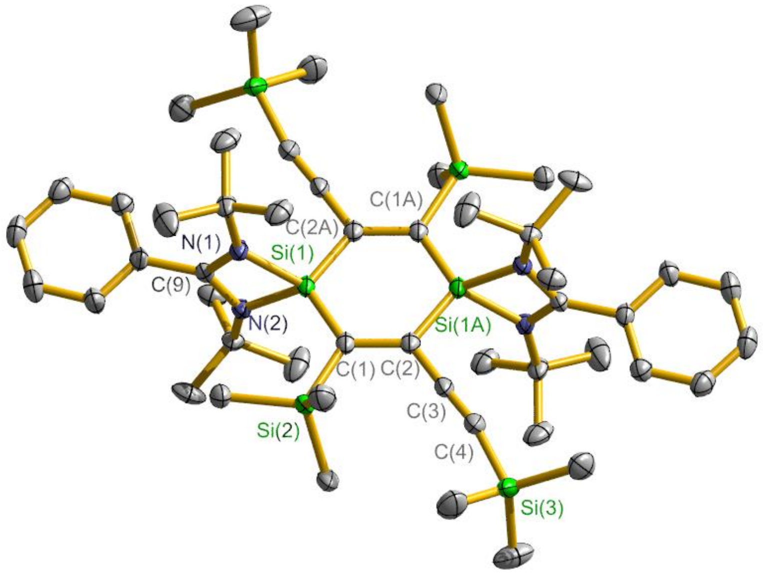

Figure S8. X-ray crystal structure of [**1**]$^{+\cdot}$ in **7** with thermal ellipsoids at 50% probability level. The hydrogen atoms are omitted for clarity. Selected bond lengths (Å) and angles (°): Si(1)–C(1) 1.800(2), Si(1)–C(2A) 1.834(2), C(1)–C(2) 1.414(3), C(2)–C(3) 1.424(3), C(3)–C(4) 1.220(4), C(4)–Si(3) 1.842(3), Si(1)–N(1) 1.8099(18), Si(1)–N(2) 1.8129(18), C(9)–N(1) 1.344(3), C(9)–N(2) 1.339(3); C(1)–Si(1)–C(2A) 116.16(10), Si(1)–C(1)–C(2) 119.06(17), C(1)–C(2)–Si(1A) 124.76(18), C(1)–C(2)–C(3) 121.8(2), C(2)–C(3)–C(4) 178.2(3), C(3)–C(4)–Si(3) 169.6(2), N(1)–Si(1)–N(2) 72.53(8), N(1)–C(9)–N(2) 106.03(17).

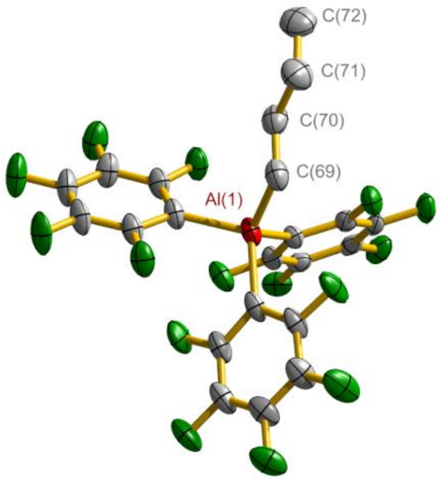

Figure S9. X-ray crystal structure of $[n\mathrm{BuAl}(C_6F_5)_3]^-$ in **7** with thermal ellipsoids at 50% probability level. The hydrogen atoms are omitted for clarity.

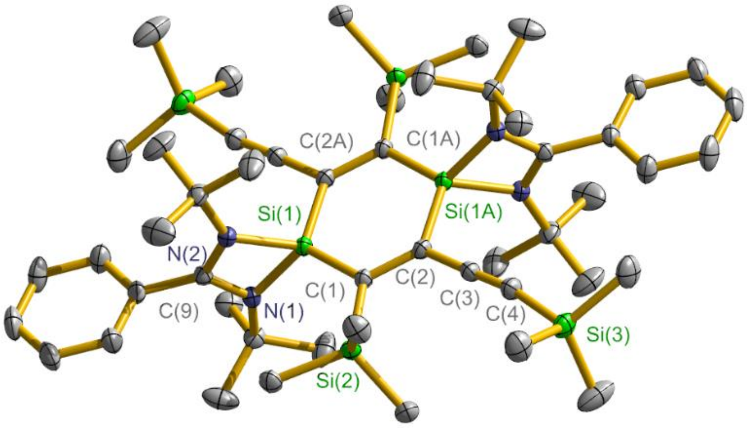

Figure S10. X-ray crystal structure of $[\mathbf{1}]^{+\cdot}$ in **8** with thermal ellipsoids at 50% probability level. The hydrogen atoms are omitted for clarity. Selected bond lengths (Å) and angles (°): Si(1)–C(1) 1.806(3), Si(1)–C(2A) 1.840(3), C(1)–C(2) 1.408(4), C(2)–C(3) 1.428(4), C(3)–C(4) 1.215(4), C(4)–Si(3) 1.844(3), Si(1)–N(1) 1.811(2), Si(1)–N(2) 1.811(2), C(9)–N(1) 1.337(3), C(9)–N(2) 1.346(3); C(1)–Si(1)–C(2A) 116.12(12), Si(1)–C(1)–C(2) 118.74(19), C(1)–C(2)–Si(1A) 125.0(2), C(1)–C(2)–C(3) 121.4(2), C(2)–C(3)–C(4) 178.3(3), C(3)–C(4)–Si(3) 169.9(3), N(1)–Si(1)–N(2) 72.36(10), N(1)–C(9)–N(2) 105.6(2).

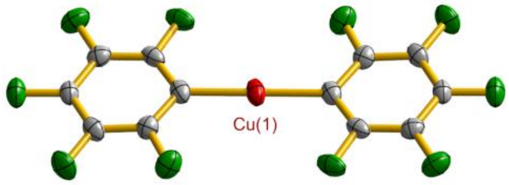

Figure S11. X-ray crystal structure of $[Cu(C_6F_5)_2]^-$ in **8** with thermal ellipsoids at 50% probability level.

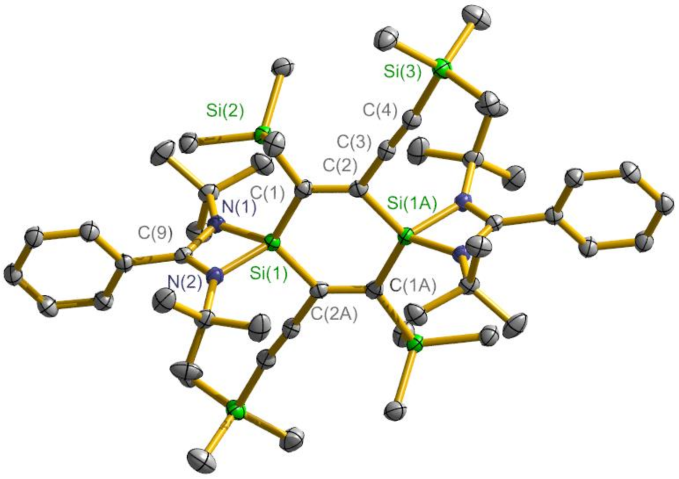

Figure S12. X-ray crystal structure of [**1**]$^{2+}$ in **9** with thermal ellipsoids at 50% probability level. The hydrogen atoms are omitted for clarity. Selected bond lengths (Å) and angles (°): Si(1)–C(1) 1.8465(15), Si(1)–C(2A) 1.8764(15), C(1)–C(2) 1.371(2), C(2)–C(3) 1.436(2), C(3)–C(4) 1.212(2), C(4)–Si(3) 1.8630(17), Si(1)–N(1) 1.7817(12), Si(1)–N(2) 1.7817(12), C(9)–N(1) 1.3466(18), C(9)–N(2) 1.3505(18); C(1)–Si(1)–C(2A) 116.32(7), Si(1)–C(1)–C(2) 117.88(11), C(1)–C(2)–Si(1A) 125.75(11), C(1)–C(2)–C(3) 122.16(13), C(2)–C(3)–C(4) 177.53(16), C(3)–C(4)–Si(3) 172.83(14), N(1)–Si(1)–N(2) 74.02(6), N(1)–C(9)–N(2) 105.47(12).

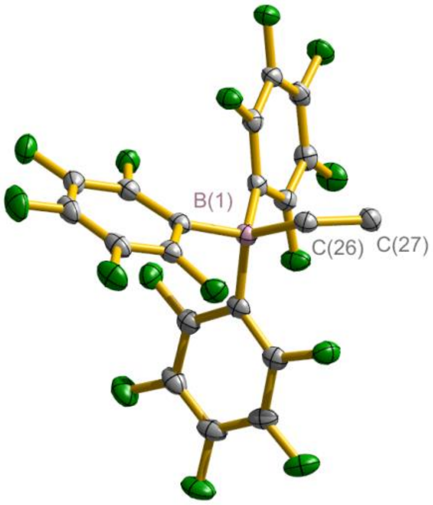

Figure S13. X-ray crystal structure of $[EtB(C_6F_5)_3]^-$ in **9** with thermal ellipsoids at 50% probability level. The hydrogen atoms are omitted for clarity.

Selected bond lengths (Å) and angles (°) for [**1**]$^{2+}$ in **10**: Si(1)–C(1) 1.832(2), Si(1)–C(2A) 1.874(2), C(1)–C(2) 1.368(3), C(2)–C(3) 1.435(3), C(3)–C(4) 1.209(4), C(4)–Si(3) 1.861(3), Si(1)–N(1) 1.777(2), Si(1)–N(2) 1.779(2), C(11)–N(1) 1.340(3), C(11)–N(2) 1.353(3); C(1)–Si(1)–C(2A) 117.32(11), Si(1)–C(1)–C(2) 117.28(18), C(1)–C(2)–Si(1A) 125.37(18), C(1)–C(2)–C(3) 122.6(2), C(2)–C(3)–C(4) 176.7(3), C(3)–C(4)–Si(3) 173.3(2), N(1)–Si(1)–N(2) 74.49(10), N(1)–C(11)–N(2) 106.1(2).

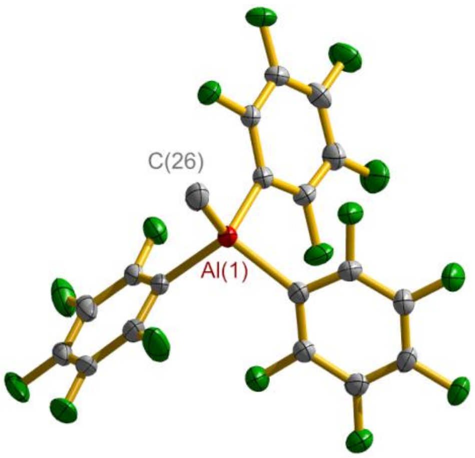

Figure S14. X-ray crystal structure of $[MeAl(C_6F_5)_3]^-$ in **10** with thermal ellipsoids at 50% probability level. The hydrogen atoms are omitted for clarity.

Selected bond lengths (Å) and angles (°) for **11**: S(1)–O(1) 1.5125(11), S(1)–O(2) 1.5187(10), S(1)–O(3) 1.476(3), S(1)–O(4) 1.387(3), Si(1)–O(1) 1.7893(11), Si(2)–O(2) 1.7983(10), Si(1)–C(1) 1.8768(15), Si(1)–C(6) 1.8982(14), Si(2)–C(2) 1.8970(15), Si(2)–C(5) 1.8821(15), C(1)–Si(3) 1.8999(14), C(1)–C(2) 1.365(2), C(2)–C(3) 1.440(2), C(3)–C(4) 1.209(2), C(4)–Si(4) 1.8309(16), C(5)–Si(5) 1.8980(15), C(5)–C(6) 1.368(2), C(6)–C(7) 1.439(2), C(7)–C(8) 1.211(2), C(8)–Si(6) 1.8426(16), Si(1)–N(1) 1.8140(12), Si(1)–N(2) 1.9942(12), C(13)–N(1) 1.3636(19), C(13)–N(2) 1.3067(19), Si(2)–N(3) 1.8153(12), Si(2)–N(4) 1.9967(12), C(28)–N(3) 1.3605(19), C(28)–N(4) 1.3104(19); O(1)–S(1)–O(2) 106.66(6), Si(1)–O(1)–S(1) 140.37(7), Si(2)–O(2)–S(1) 137.54(7), C(1)–Si(1)–C(6) 115.84(6), Si(1)–C(6)–C(5) 122.90(11), C(6)–C(5)–Si(2) 116.84(11), C(5)–Si(2)–C(2) 115.33(6), Si(2)–C(2)–C(1) 123.78(11), C(2)–C(1)–Si(1) 116.22(11), Si(1)–C(1)–Si(3) 122.81(8), C(1)–C(2)–C(3) 122.05(13), C(2)–C(3)–C(4) 176.30(15), C(3)–C(4)–Si(4) 170.84(14), Si(2)–C(5)–Si(5) 121.07(8), C(5)–C(6)–C(7) 121.76(13), C(6)–C(7)–C(8) 174.15(15), C(7)–C(8)–Si(6) 167.17(14), N(1)–Si(1)–N(2) 68.45(5), N(1)–C(13)–N(2) 107.02(12), N(3)–Si(2)–N(4) 68.48(5), N(3)–C(28)–N(4) 107.22(12).

III. Selected EPR spectra

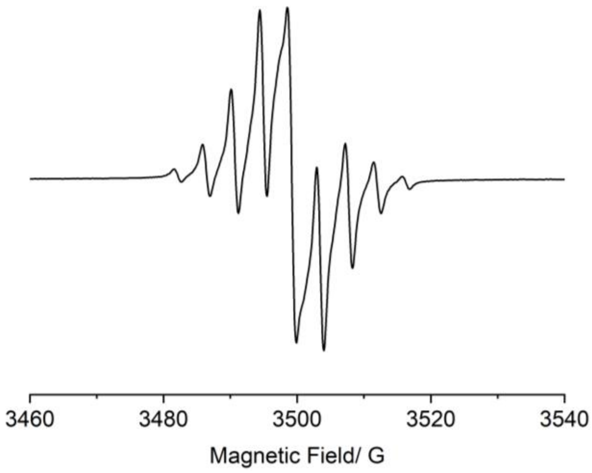

Figure S15. EPR spectrum of compound **2** in toluene at room temperature.

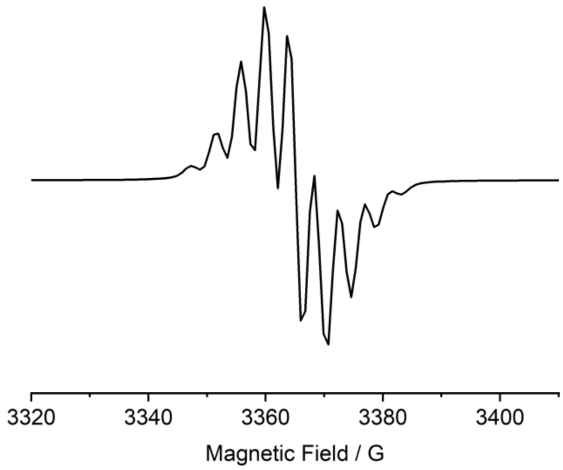

Figure S16. EPR spectrum of compound **3** in toluene at room temperature.

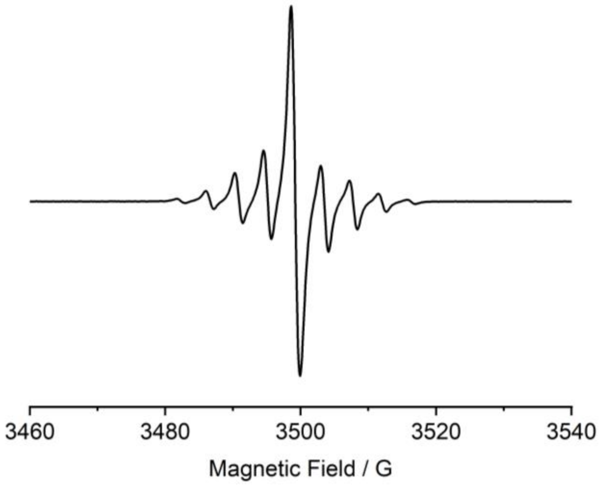

Figure S17. EPR spectrum of compound **4** in toluene at room temperature.

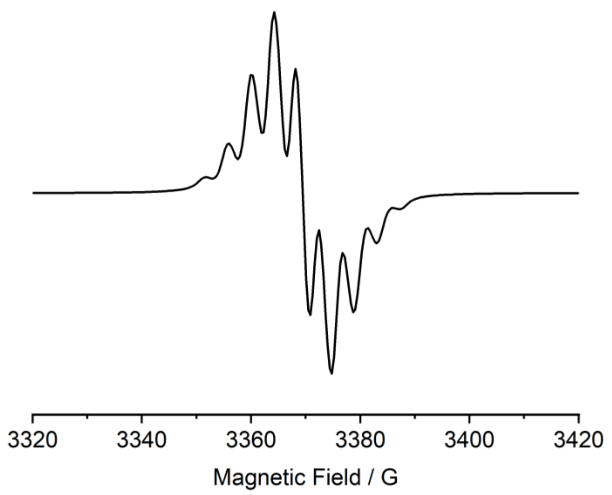

Figure S18. EPR spectrum of compound **5** in toluene at room temperature.

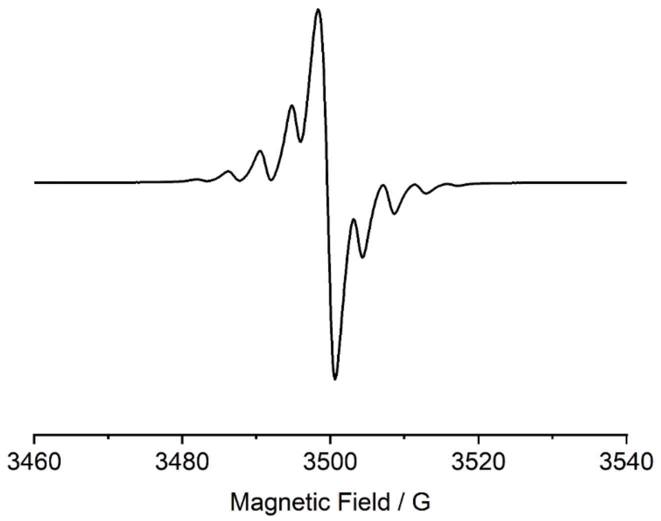

Figure S19. EPR spectrum of compound **7** in toluene at room temperature.

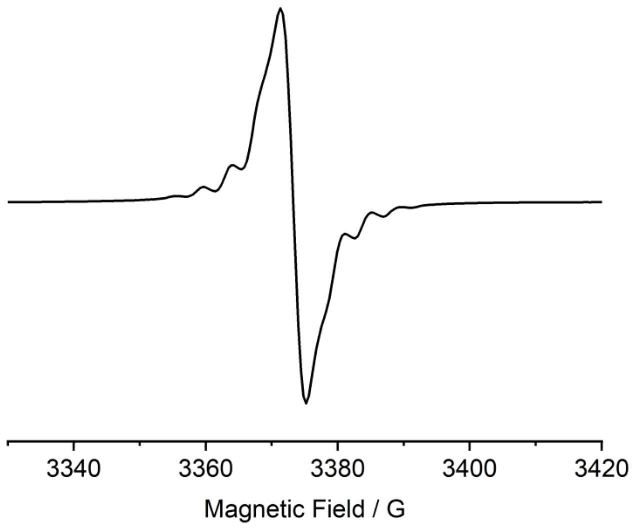

Figure S20. EPR spectrum of compound **8** in toluene at room temperature.

The EPR spectra of complexes **2**–**8** were each measured in toluene at 298 K, which all exhibited well-resolved signals consisting of nine resonances (Figures 1 and S15–S20). These signals are centered at a close *g* value of 2.0038 due to the same [**1**]$^{+\cdot}$ cation in each of **2**–**8**. The generation of nine resonances is due to hyperfine coupling with four chemically equivalent nitrogen nuclides (a(^{14}N) = 4.05 G) from the two L ligands chelated at the respective two Si atoms of the C_4Si_2 ring. The coupling to the silicon nucleus is not observed due to the low natural abundance (4.67%) of the ^{29}Si nuclide. These data suggest that delocalization of the one-electron is over the central C_4Si_2 ring of [**1**]$^{+\cdot}$, rather than at one of the two Si atoms therein, in each of **2**–**8**. This is in agreement with the Mulliken spin density data previously computed, where the unpaired electron mainly equally delocalizes over the two C_2Si moieties of the C_4Si_2 cycle and the spin density is Si 0.12, C 0.12, and 0.14 e, respectively.[S3] It is noted that the EPR spectra of **2**–**8** exhibit resonances distinguishable from each other. This implies different electronic statistic interactions occurred for the different anions (**2**–**8**) with [**1**]$^{+\cdot}$ (Figures S2, S4, S6–S7, S9, S11, and S13–S14).

Reference

(S3) Y. Chen, J. Li, Y. Zhao, L. Zhang, G. Tan, H. Zhu, H. W. Roesky, *J. Am. Chem. Soc*. **2021**, *143*, 2212−2216.

IV. Selected UV-vis absorption spectra

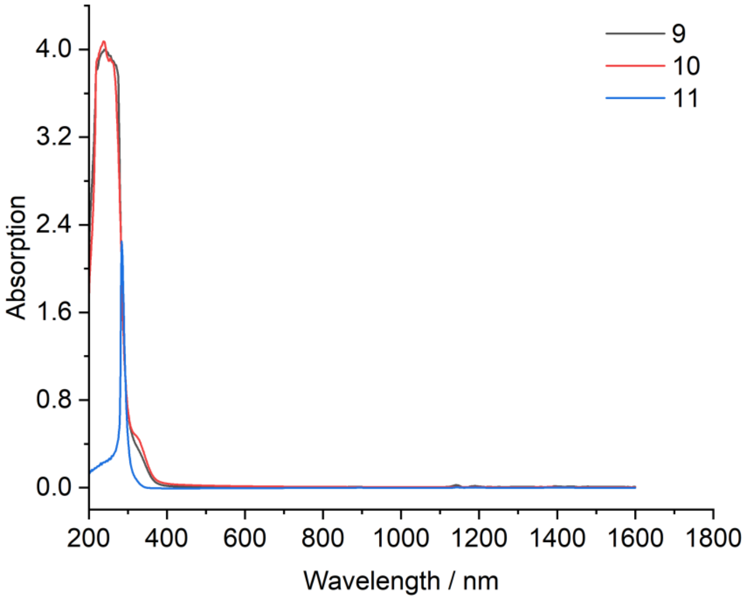

Figure S21. UV-Vis absorption spectra of compounds **9**–**10** in CH_3CN and **11** in toluene all at room temperature

V. Collected ^{1}H, ^{13}C, ^{19}F, and ^{29}Si NMR spectra of compounds 9−11

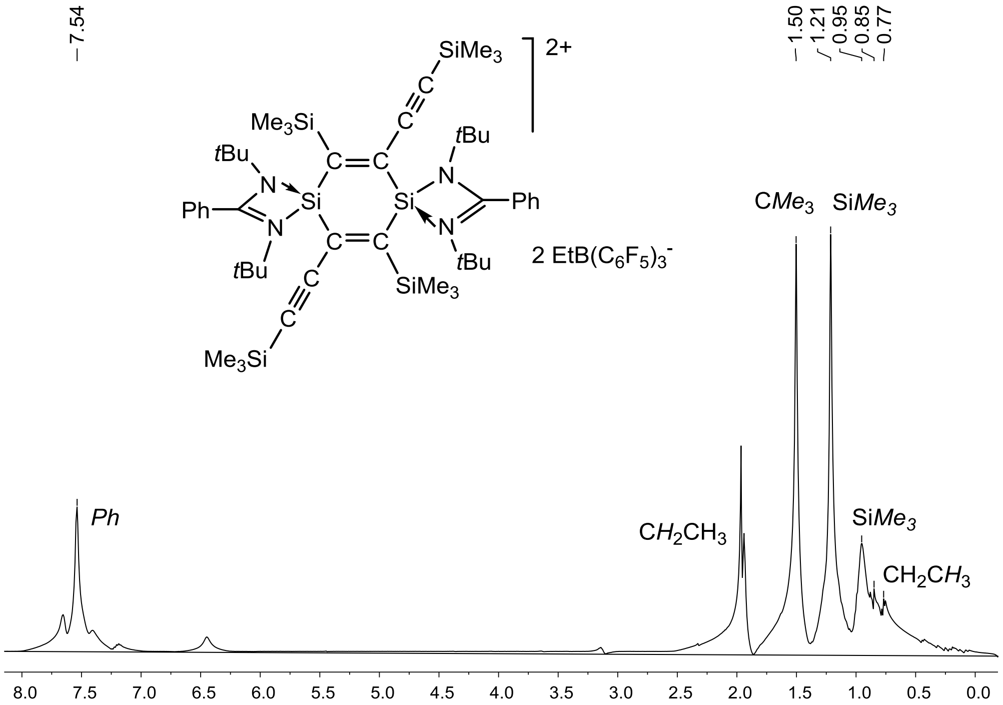

Figure S22. ^{1}H NMR spectrum of compound **9** in CD_3CN at 298 K

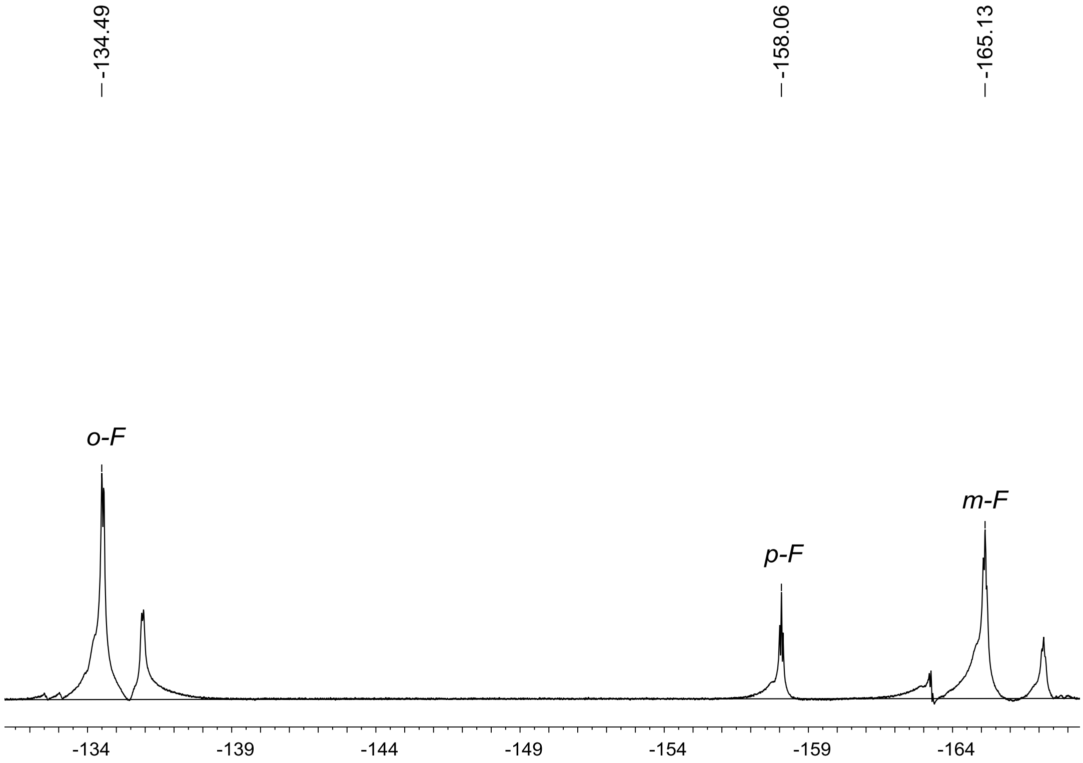

Figure S23. ^{19}F NMR spectrum of compound **9** in CD_3CN at 298 K (another set of three resonances is from $B(C_6F_5)_3$ unreacted)

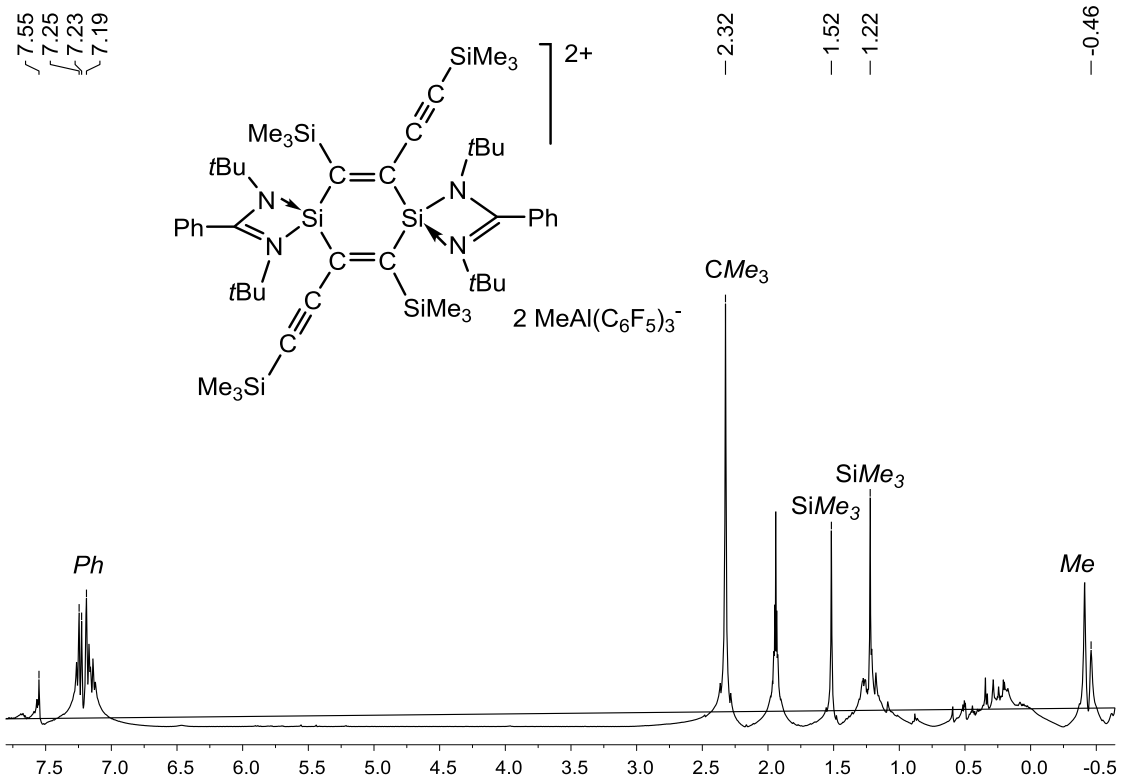

Figure S24. ^{1}H NMR spectrum of compound **10** in CD_3CN at 298 K

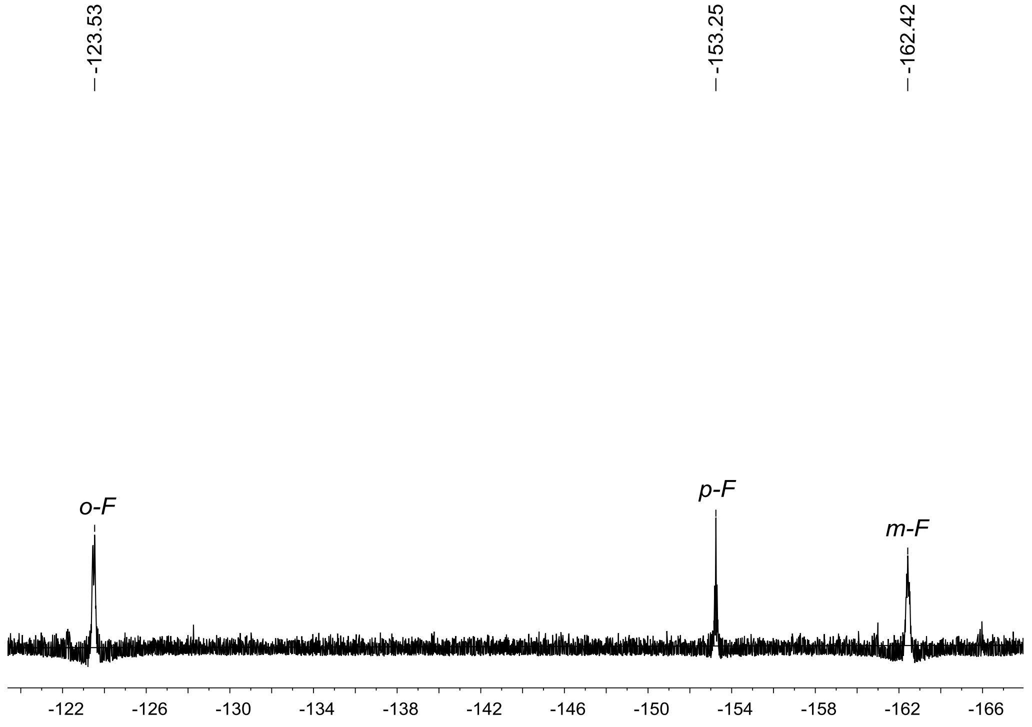

Figure S25. ^{19}F NMR spectrum of compound **10** in CD_3CN at 298 K

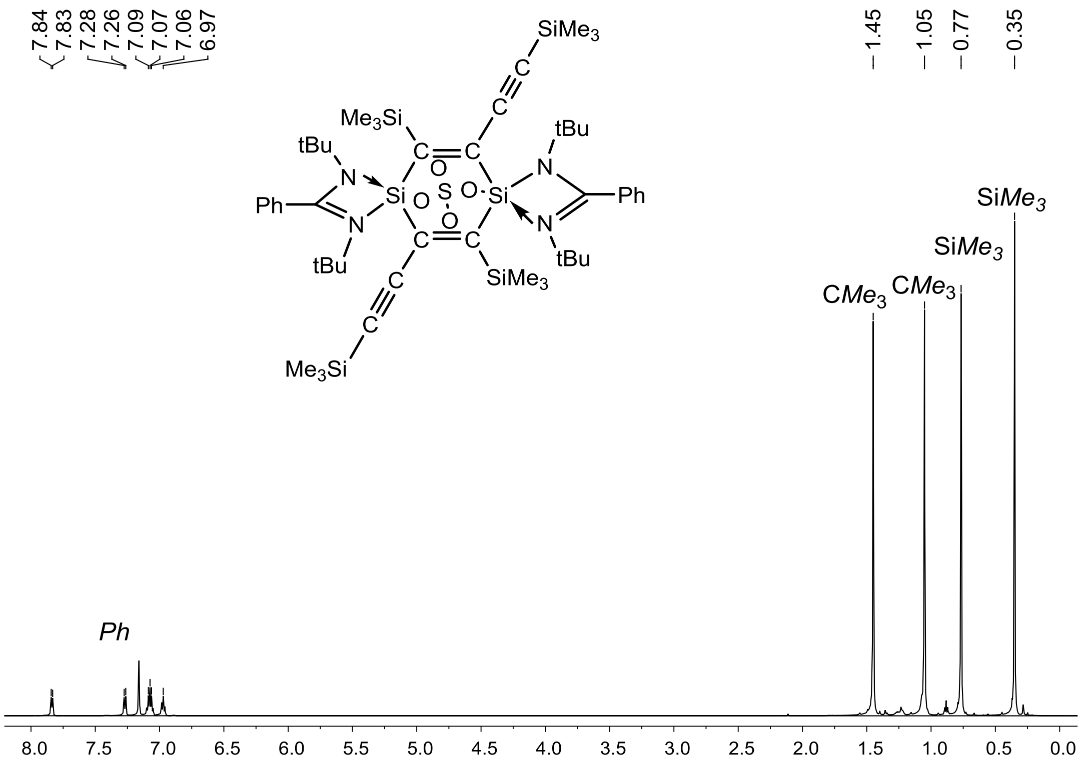

Figure S26. ^{1}H NMR spectrum of compound **11** in C_6D_6 at 298 K

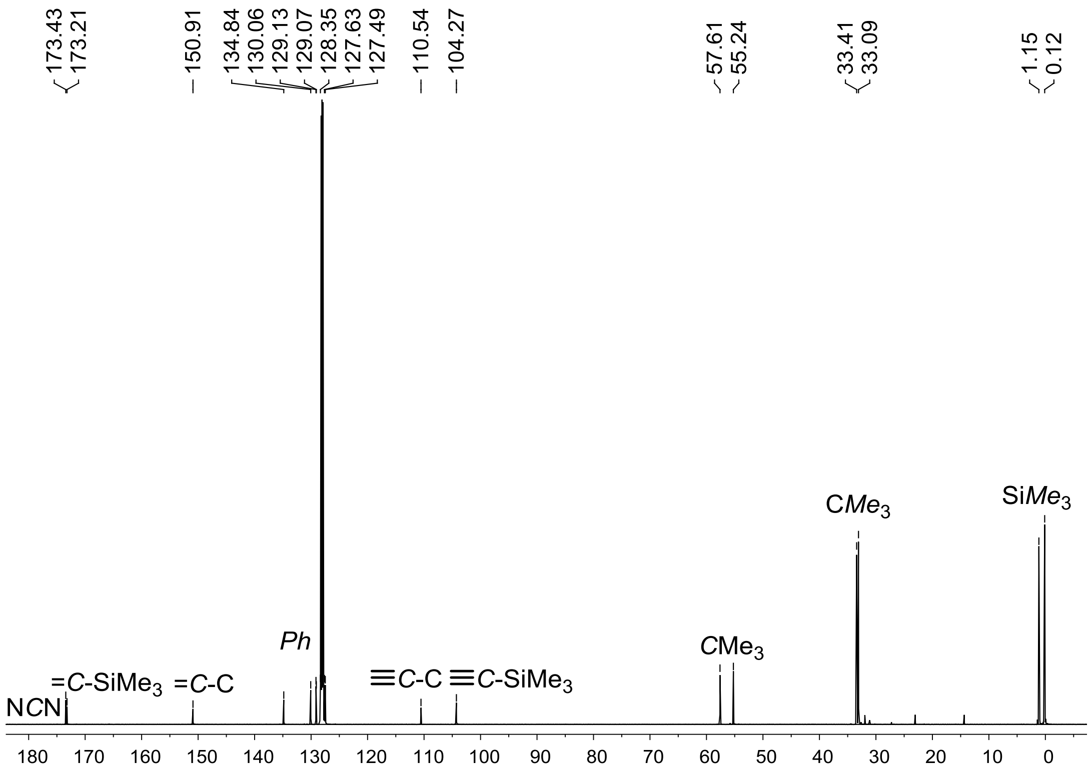

Figure S27. ^{13}C NMR spectrum of compound **11** in C_6D_6 at 298 K

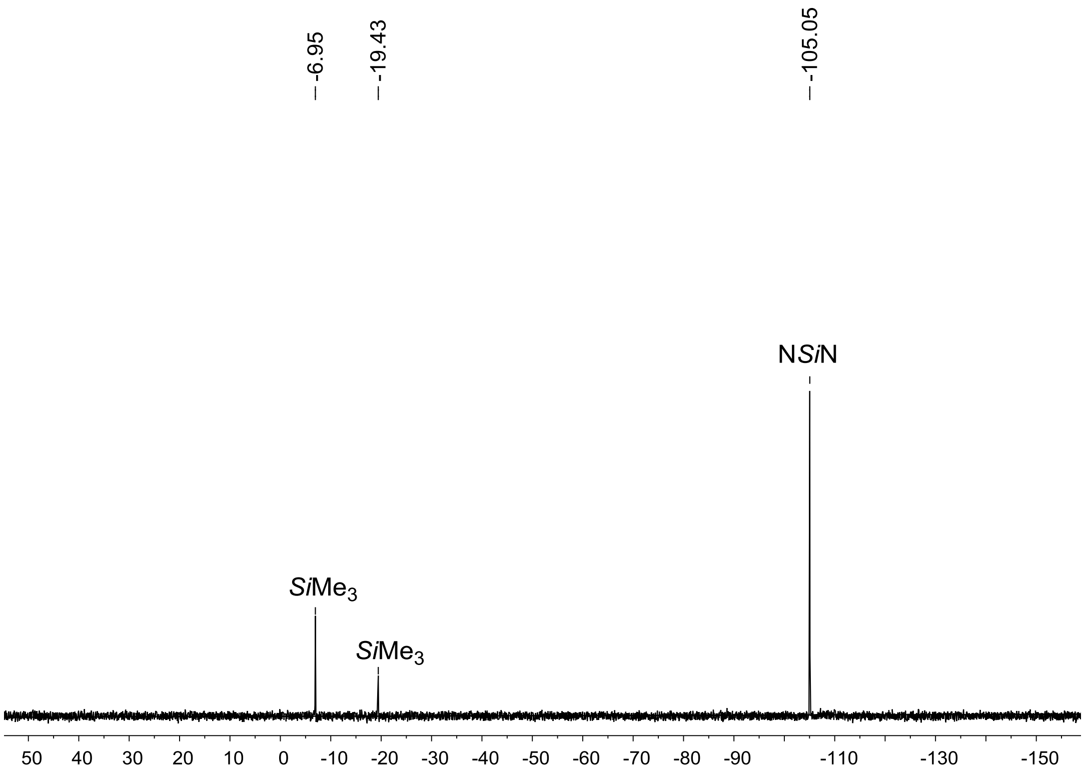

Figure S28. ^{29}Si NMR spectrum of compound **11** in C_6D_6 at 298 K

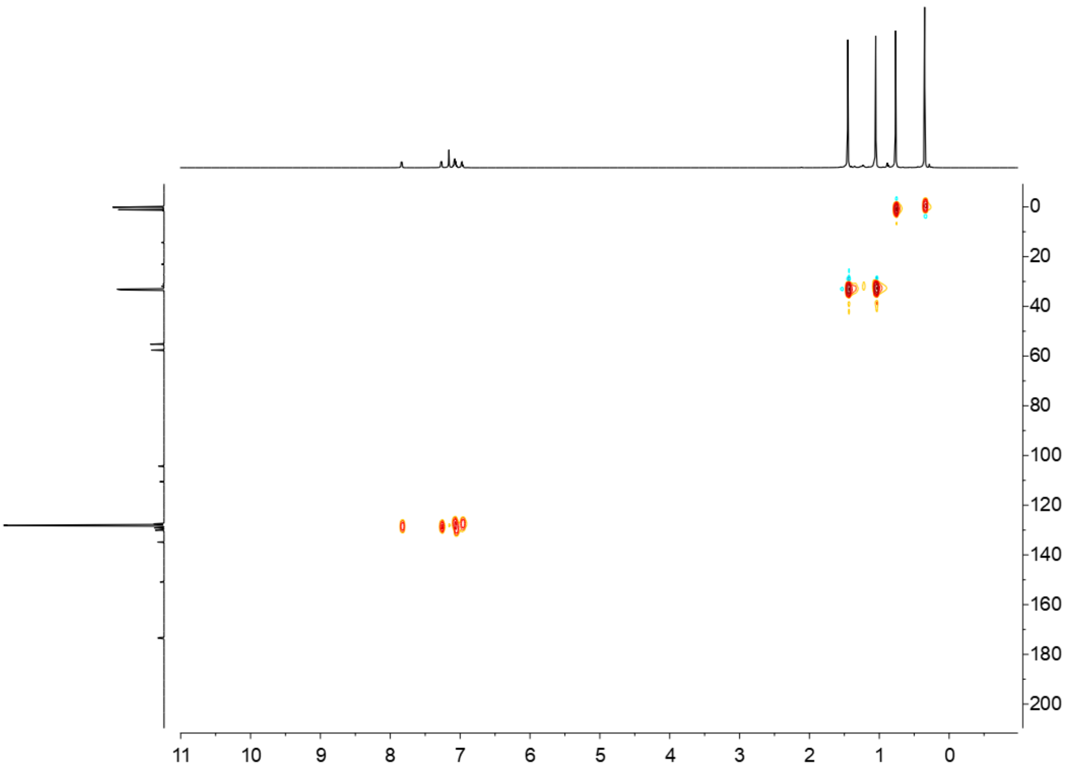

Figure S29. ^{1}H,^{13}C-HSQC spectrum of compound **11** in C_6D_6 at 298 K

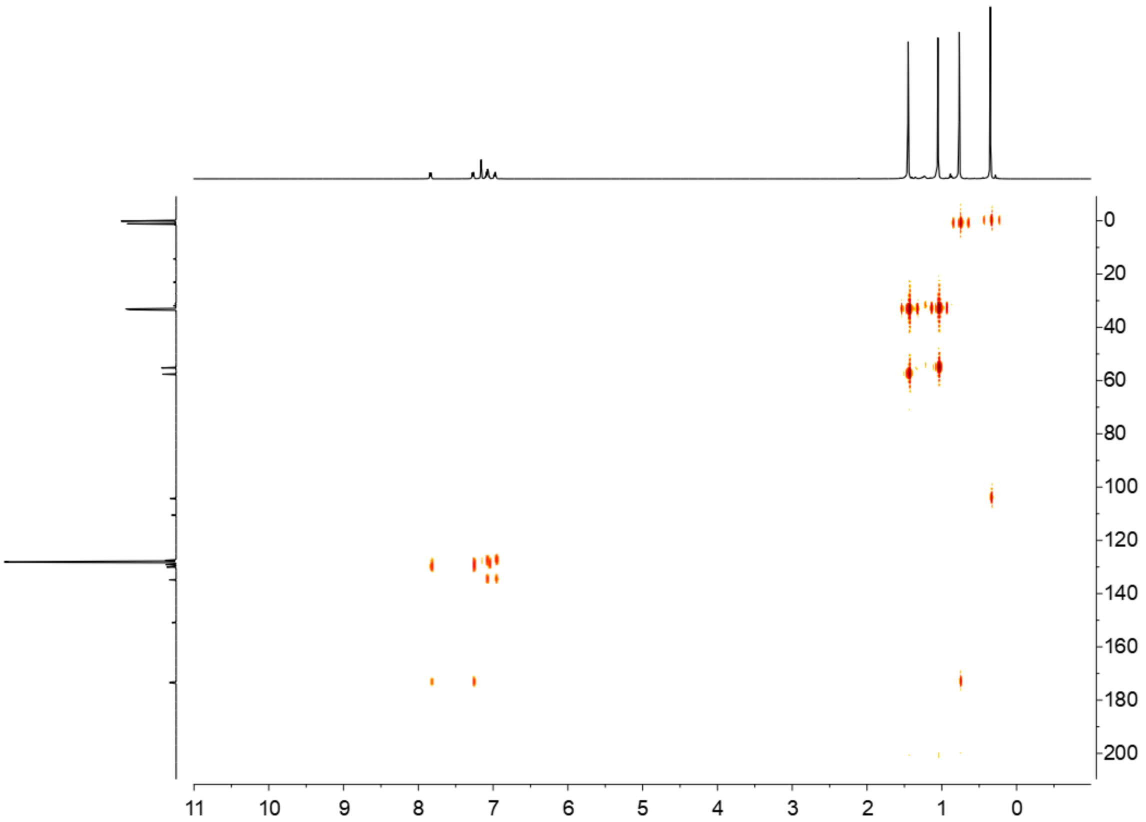

Figure S30. $^{1}H,^{13}C$-HMBC spectrum of compound **11** in C_6D_6 at 298 K

Due to the solubility issue, the ^{1}H, ^{13}C, and ^{29}Si NMR data were gained for compound **11** while the ^{1}H and ^{19}F spectra were afforded for compounds **9–10.** The ^{29}Si NMR spectrum of **11** shows three resonances. The resonance at δ –105.05 ppm is assignable to the Si nucleus from the C_4Si_2-ring, which is comparable to those found in the same ring of $[\mathbf{1}]^{2+}$ in $[\mathbf{1}]^{2+}$ $2[OSO_2CF_3]^-$ (δ –105.84 ppm)[S3] and of $[(L)_2Si_2C_4(SiMe_3)_2(C_2SiMe_3)_2](\mu\text{-}O_2)$ (δ –94.42 ppm), but significantly different from that in the C_4Si_2-ring of **1** (δ –8.36 ppm) due to two more electron delocalization.[S4] The resonances at the respective δ –19.43 and –6.95 ppm are due to the two *Si*Me_3 having different structural environments. The combined ^{1}H and ^{13}C NMR data indicate the two Si*Me*$_3$ ($\delta_{H(Me)}$ 0.35 and $\delta_{C(Me)}$ 0.12 ppm for the $SiMe_3$ bonded to the C=C, and $\delta_{H(Me)}$ 0.77 and $\delta_{C(Me)}$ 1.05 ppm for the $SiMe_3$ to the C≡C) groups. Meanwhile, the *tBu* moieties at the two L ligands are also confirmed ($\delta_{H(CMe3)}$ 1.05 and 1.45 and $\delta_{C(CMe3)}$ 33.09 and 33.41 and $\delta_{C(CMe3)}$ 55.24 and 57.61 ppm). The ^{13}C NMR data clearly disclose the C=C (δ 150.91 and 173.21 ppm) functionality inside the C_4Si_2-ring while the C≡C (δ 104.27 and 110.54 ppm) group are outside. For comparison, the Si*Me*$_3$ (δ 0.95 and 1.21 ppm for **9**; δ 1.22 and 1.27 ppm for **10**) and *tBu* (δ 1.50 ppm for **9** and δ 2.32 ppm for **10**) groups are able to identify from their ^{1}H NMR spectra. In addition, the ^{19}F NMR spectrum of **9** (in CD_3CN) displays three resonances at δ –134.49 (*o-F*), –158.06 (*p-F*), and –165.13 (*m-F*) ppm, assigned to the C_6F_5 group from the $[EtB(C_6F_5)_3]^-$ and the ^{1}H NMR spectrum indicates the Et group by showing the resonances at δ 0.85 (C*H*$_3$) and 1.95 (C*H*$_2$) ppm. These data are comparable to those found in $[EtB(C_6F_5)_3]^-$ of $(Ph_3C)^+[EtB(C_6F_5)_3]^-$ (in C_6D_6, δ_F –136.74 (*o-F*), –160.26 (*p-F*), –163.58 (*m-F*) and δ_H 0.88 (C*H*$_3$), 2.0 (C*H*$_2$) ppm).[S5] Similarly, the $[MeAl(C_6F_5)_3]^-$ in **10** is confirmed from the ^{19}F (in CD_3CN, δ –123.53 (*o-F*), –153.25 (*p-F*), and –162.42 (*m-F*) ppm) and ^{1}H (δ –0.46 (*Me*) ppm) NMR data. Comparable data are observed for the same anion in $(TaCp^*Me_3)^+[MeAl(C_6F_5)_3]^-$ (in CD_2Cl_2, δ_F –119.0 (*o-F*), –156.5 (*p-F*), and –161.5 (*m-F*) and δ_H –0.44 (*Me*) ppm).[S6]

References

(S4) Y. Chen, J. Li, W. Jiang, J. Zhao, H. Zhu, S. Muhammed, P. Parameswaran, H. W. Roesky, *Organometallics* **2020**, *39*, 4282–4286.

(S5) a) N. Bavarian, M. C. Baird, *Organometallics* **2005**, *24*, 2889–2897; b) J. Sánchez-Nieves, P. Royo, *Organometallics* **2007**, *26*, 2880–2884.

(S6) E. Ihara, V. G. Young, Jr., R. F. Jordan, *J. Am. Chem. Soc*. **1998**, *120*, 8277–8278.

END
